# Semaglutide reduces tumor burden in the GAN diet-induced obese and biopsy-confirmed mouse model of NASH-HCC with advanced fibrosis

**DOI:** 10.1038/s41598-023-50328-5

**Published:** 2023-12-27

**Authors:** Henrik H. Hansen, Susanne Pors, Maja W. Andersen, Mogens Vyberg, Jacob Nøhr-Meldgaard, Malte Hasle Nielsen, Denise Oró, Martin Rønn Madsen, Monika Lewinska, Mathias B. Møllerhøj, Andreas Nygaard Madsen, Michael Feigh

**Affiliations:** 1 Gubra, Hørsholm Kongevej 11B, DK-2970 Hørsholm, Denmark; 2 Center for RNA Medicine, Department of Clinical Medicine, Aalborg University, Copenhagen, Denmark

**Keywords:** Pharmacology, Cancer, Metabolic disorders

## Abstract

Non-alcoholic steatohepatitis (NASH) is emerging as a major cause of hepatocellular carcinoma (HCC), however, it is not resolved if compounds in late-stage clinical development for NASH may have additional therapeutic benefits in NASH-driven HCC (NASH-HCC). Here, we profiled monotherapy with semaglutide (glucagon-like-receptor-1 receptor agonist) and lanifibranor (pan-peroxisome proliferator-activated receptor agonist) in a diet-induced obese (DIO) mouse model of NASH-HCC. Disease progression was characterized in male C57BL/6 J mice fed the GAN (Gubra Amylin NASH) diet high in fat, fructose and cholesterol for 12–72 weeks (n = 15 per group). Other GAN DIO-NASH-HCC mice fed the GAN diet for 54 weeks and with biopsy-confirmed NASH (NAFLD Activity Score ≥ 5) and advanced fibrosis (stage F3) received vehicle (n = 16), semaglutide (30 nmol/kg, s.c., n = 15), or lanifibranor (30 mg/kg, p.o., n = 15) once daily for 14 weeks. GAN DIO-NASH-HCC mice demonstrated progressive NASH, fibrosis and HCC burden. Tumors presented with histological and molecular signatures of poor prognostic HCC. Consistent with clinical trial outcomes in NASH patients, both lanifibranor and semaglutide improved NASH while only lanifibranor reduced fibrosis in GAN DIO-NASH-HCC mice. Notably, only semaglutide reduced tumor burden in GAN DIO-NASH-HCC mice. In conclusion, the GAN DIO-NASH-HCC mouse is a clinical translational model of NASH-HCC. Semaglutide improves both NASH and tumor burden in GAN DIO-NASH-HCC mice, highlighting the suitability of this preclinical model for profiling novel drug therapies targeting NASH-HCC.

## Introduction

Non-alcoholic steatohepatitis (NASH) is the most severe form of non-alcoholic fatty liver disease (NAFLD), the most common chronic liver condition worldwide^1^. NASH is characterized by steatosis, lobular inflammation and hepatocyte ballooning degeneration^2^. NASH patients are at increased risk for developing liver fibrosis, the strongest predictor for severe complications, notably cirrhosis, hepatocellular carcinoma (HCC) and end-stage liver disease. HCC, accounting for ~ 90% of primary liver cancers, is the fourth-leading cause of cancer-related mortality worldwide^3,4^. Mortality in NASH-HCC patients is comparable with other causes of HCC^5^. While development of cirrhosis is a major risk factor for NASH-driven HCC (NASH-HCC), the occurrence of NASH-HCC in non-cirrhotic patients is increasingly recognized^6,7^. Fueled by the obesity and diabetes epidemics^8^, NASH has arisen to become the fastest growing cause of HCC and set to become the leading etiology for HCC in 2030^9^. Treatment options for advanced HCC are very limited. Furthermore, there is an increasing appreciation that efficacy of current HCC-targeted immunotherapies might be affected by different underlying liver disease etiologies^10,11^. Accordingly, preliminary clinical data have indicated better immunotherapy outcomes in HCC with viral compared to nonviral etiology^10^. As result, NASH has recently been suggested to be a predictor of unfavourable outcome in patients treated with immune-checkpoint inhibitors^12,13^. In support, preclinical studies have indicated reduced efficacy of HCC immunotherapies in NASH-HCC mouse models compared to non-NASH HCC mouse models^12,14,15^, inviting the possibility that NASH may be an immunologically distinct pro-tumorigenic liver disease.

The alarming trend and poor prognosis associated with NASH‐HCC emphasizes the high unmet need for effective treatments for NASH and NASH-HCC. While several animal models of NASH-HCC are used in preclinical target and drug discovery for HCC, many do not faithfully reproduce the human disease^16,17^. Consequently, there is an increasing need for improved clinical translational models of NASH-HCC that can help advancing drug candidates from preclinical to clinical drug development. The Gubra-Amylin NASH (GAN) diet-induced obese (DIO) mouse model of biopsy-confirmed NASH (GAN DIO-NASH mouse) is an industry-standard translational model of NASH, recapitulating the natural history and hallmarks of NASH with progressive fibrosis^18,19^. Interestingly, GAN DIO-NASH mice demonstrate high incidence of liver tumors resembling morphological and histological features of human HCC following extended GAN diet feeding^20^. The present study therefore aimed to perform a detailed characterization of disease progression, tumor burden, and HCC histological and molecular classification in GAN DIO-NASH mice.

While effective drug therapies are emerging for management of NASH^21^, no approved medical treatments for NASH exist. Consequently, no current investigational drugs targeting NASH have been specifically evaluated for potential therapeutic effects in NASH-HCC. Nonetheless, a reduced risk of HCC has been reported in NASH patients undergoing bariatric surgery^22^, which is a hopeful indication that pharmacological interventions effective in NASH might also improve outcomes in NASH-HCC patients. Semaglutide, a long-acting glucagon-like receptor 1 (GLP1R) agonist currently approved for treatment of type 2 diabetes and obesity^23,24^, has been reported to increase resolution of NASH without improving fibrosis stage in a proof-of-concept clinical trial^25^. In comparison, lanifibranor, a pan-peroxisome proliferator-activated receptor (PPAR-α/δ/γ) agonist, achieved significant benefits on both NASH and fibrosis histology in a recent clinical phase 2b study (NATIVE trial)^26^. In the present study, we therefore also asked if semaglutide and lanifibranor could have therapeutic efficacy on NASH and tumor burden in GAN DIO-NASH-HCC mice.

## Results

### GAN DIO-NASH-HCC mice recapitulates the natural history of NASH-HCC

**Figure 1:**
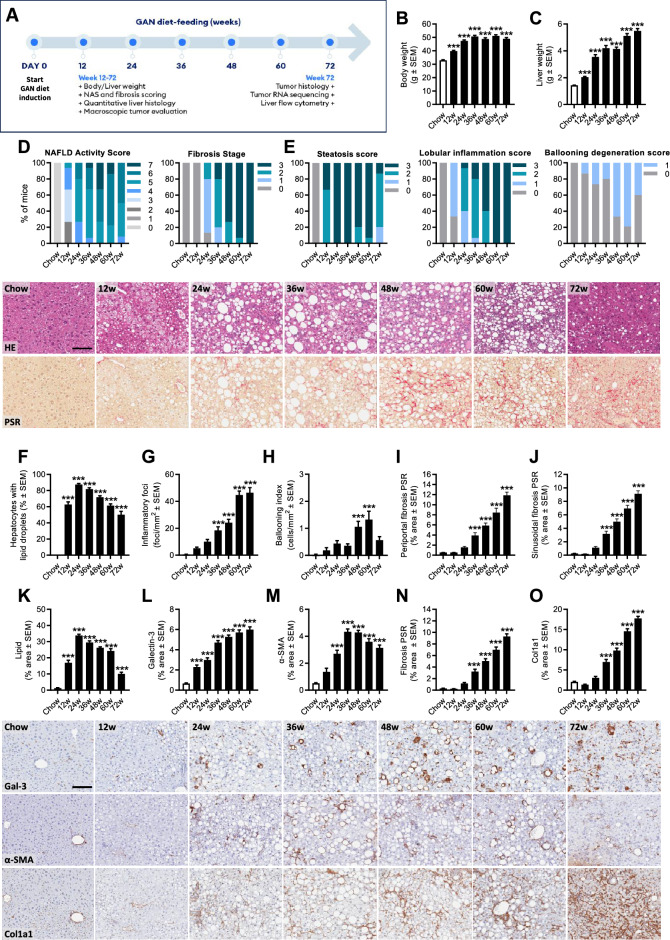
GAN DIO-NASH-HCC mice show progressive development of severe NASH with advanced fibrosis. Mice were fed the GAN diet for 12–72 weeks (n = 15 per group). Chow-fed mice (n = 10) served as normal controls. (**A**) Study outline illustrating endpoints applied at all GAN diet-induction periods (plasma/liver biochemistry, NAS and fibrosis scoring, quantitative liver histology, liver (non-tumor) RNA sequencing, macroscopic tumor counts) and additional endpoints applied after 72 weeks of GAN diet feeding (tumor histology, tumor RNA sequencing, liver flow cytometry). (**B**) Terminal body weight. (**C**) Liver weight. (**D**) NAFLD Activity Score (NAS) and fibrosis stage. (**E**) Steatosis, lobular inflammation and ballooning degeneration scores. (**F–J**) GHOST-based histomorphometrics on histopathological scoring variables, including lipid-laden (**F**) hepatocytes, (**G**) inflammatory foci, (**H**) hepatocyte ballooning and (**I**, **J**) periportal/sinusoidal fibrosis. Lower panels: HE and PSR stainings illustrating the development of steatosis and perisinusoidal fibrosis in GAN DIO-NASH-HCC mice (scale bar, 100 µm). (**K–O**) Proportionate (%) area of (**K**) lipids, (**L**) inflammation (galectin-3), (**M**) α-SMA (fibrogenesis marker) and (**N**, **O**) fibrosis (PSR, Col1a1). See Fig. S1 for total liver histological marker levels. Lower panels: Representative photomicrographs of galectin-3, α-SMA, and Col1a1 immunostainings (scale bar, 100 µm). ***p < 0.001 versus chow-fed controls (Dunnett’s test one-factor linear model).

Metabolic, histological and transcriptome markers of NASH were profiled longitudinally in DIO-NASH mice fed the GAN diet for 12–72 weeks, see study outline in Fig. 1A. Weight gain in DIO-NASH mice increased progressively with extending the GAN diet feeding period compared to chow-fed mice (Fig. 1B), and was closely reflected by worsening of hepatomegaly (Fig. 1C). A deep learning-based digital imaging analysis pipeline (GHOST) was applied for automated, unbiased scoring of NAS variables and fibrosis stage. Manifest NASH (NAS 4–5) was consistently observed at 24 weeks (Fig. 1D). Moderate-severe steatosis was evident from 12 weeks and all mice demonstrated advanced steatosis (score 3) from 24 to 36 weeks followed by a gradual decline in NAS explained by reduced steatosis score from 48 weeks and onwards (Figs. 1E, S1). Lobular inflammation increased in severity during the whole study period with all mice demonstrating severe inflammation (score 3) at 72 weeks (Fig. 1E). When present, ballooning degeneration was mild (score 1, Fig. 1E). Hepatic fibrosis increased in severity over the course of the study with gradual transition to advanced fibrosis (stage F3, bridging fibrosis) from 36 weeks which became increasingly manifest in the cohort with extended periods of GAN diet feeding. As result, virtually all mice developed advanced fibrosis at ≥ 60 weeks of GAN diet feeding (Fig. 1D). It is noteworthy that 100% occurrence of advanced fibrosis at 72 weeks (Fig. 1D) coincided with declining steatosis score (Fig. 1E) and histomorphometric evidence of liver fat depletion (Figs. 1F, K, S1). GHOST deep learning-based image analysis also enabled quantification of histopathological scoring-derived endpoints, further emphasizing the marked dynamics in hepatocyte lipid load, inflammatory foci count, hepatocyte ballooning degeneration and periportal-sinusoidal zonation of fibrosis (Fig. 1F–J). Progressive NASH and fibrosis in GAN DIO-NASH-HCC mice was supported by quantitative histomorphometric analysis, indicating progressive increases in lipid accumulation (HE staining), inflammation (galectin-3), fibrogenic cell activity (α-SMA) and collagen deposition (PSR staining, Col1a1) (Figs. 1K–O, S1). Hepatic immune cell composition in GAN DIO-NASH-HCC mice (72 weeks of GAN diet feeding) was assessed by flow cytometry (Fig. S2). GAN DIO-NASH-HCC mice showed significant expansions in myeloid immune cell populations (CD45^+^CD11b^+^), dominated by increased number of cells with high expression of Ly6C (Ly6C^++^), a signature of infiltrating inflammatory monocytes/macrophages^27^, resident Kupffer-like macrophages (F4/80^low^CD11c^high^) and dendritic-like cells (F4/80^high^CD11c^low^)^27^, with reciprocal reductions in Ly6G^+^ neutrophils. Enhanced lymphocyte recruitment to the liver was indicated by specific accumulation of cytotoxic T-cells (CD8^+^).

### GAN DIO-NASH-HCC mice show histological and molecular signatures of poor prognostic HCC

**Figure 2:**
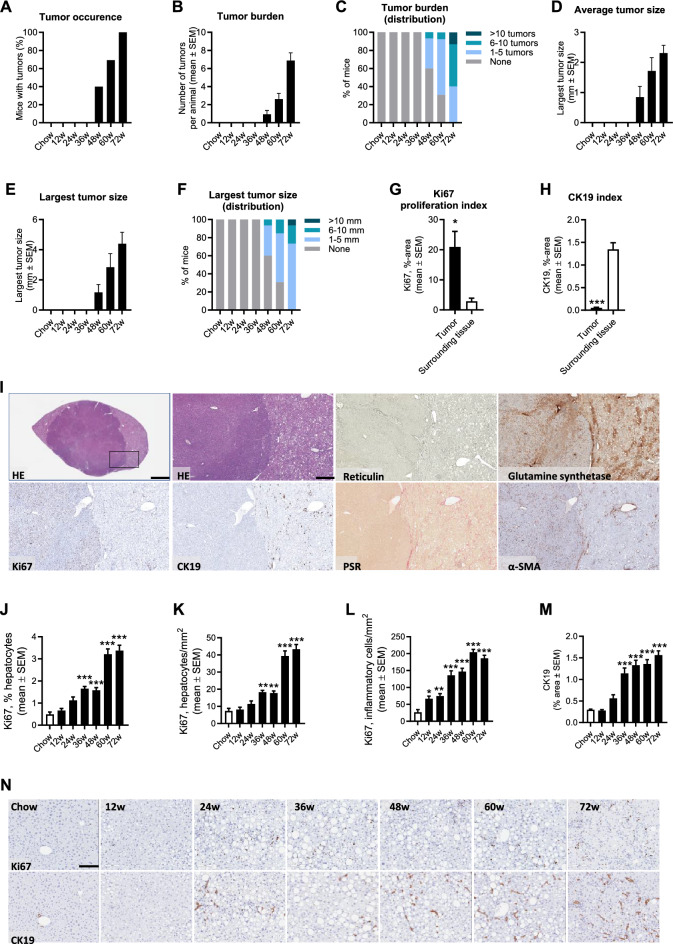
GAN DIO-NASH-HCC mice show progressive HCC burden. Mice were fed the GAN diet for 12–72 weeks (n = 15 per group). Chow-fed mice (n = 10) served as normal controls. (**A**) Tumor occurence. (**B**) Tumor burden. (**C**) Distribution of tumor burden in the cohort. (**D**) Average tumor size (mm). (**E**) Largest tumor size (diameter, mm). (**F**) Distribution of largest tumor size in the cohort. (**G**, **H**) Proportionate (%) area of Ki67 and CK19 staining in tumor (n = 5 HCCs) vs. surrounding non-tumorous tissue, *p < 0.05, ***p < 0.001 versus surrounding tissue (t-test). (**I**) Histological characteristics of liver neoplastic lesions in GAN DIO-NASH-HCC mice (≥ 68 weeks of GAN diet feeding). Insert in upper left panel (HE straining) indicates further magnified area (scale bar, 100 µm). Tumors demonstrated pushing growth and were typically devoid of lipid droplets, contrasting the surrounding liver fat-enriched tissue. Tumors demonstrated extensive or complete loss of reticulin trabecular framework and diffuse glutamine synthetase staining, being histological characteristics of HCC. A proliferating phenotype was indicated by extensive Ki67 labelleling in tumors compared to the surrounding non-tumorous tissue. Tumors showed low levels/loss of CK19-immunoreactivity, suggesting reduced/lack of biliary epithelia. Overall, tumors did not show detectable collagen deposition (PSR staining) while consistently demonstrating α-SMA immunoreactivity. (**J**–**L**) Ki67 staining of hepatocytes (relative number, %; area, mm^2^) and inflammatory cells (area, mm^2^) in non-tumorous (parenchyme) tissue. (**M**) Proportionate (%) area of parenchymal CK19 staining. (**N**) Representative immunohistochemical stainings illustrating the progressive increase in parenchymal Ki67 and CK19 staining. *p < 0.05, **p < 0.01, ***p < 0.001 vs. chow-fed controls (Dunnett’s test one-factor linear model).

Macroscopically visible hepatic neoplasms were first detected at 48 weeks with progressive cohort penetrance, resulting in 100% incidence at 72 weeks of GAN diet feeding (Fig. 2A). Correspondingly, tumor burden increased progressively over the study period as reflected by incremental increases in the number and size of tumors (Fig. 2B–F). A series of histological stainings were performed to characterize liver tumors (Fig. 2I). Tumors demonstrated pushing growth indicated by a clear compression zone between the neoplastic and normal liver tissue. Tumors were hepatocytic in nature and demonstrated cytologic atypia, including increased nuclear/cytoplasmic ratio with or without nuclear pleomorphism, and typically devoid of lipid droplets markedly contrasting the surrounding liver fat-enriched tissue. Overall, tumor lesions demonstrated lack of reticulin trabecular framework in addition to diffuse/reduced glutamine synthetase staining, representing standard diagnostic criteria for HCC^28,29^. Tumors did not show detectable collagen deposition while consistently demonstrating α-SMA immunoreactivity. Compared to surrounding tissue, tumors were clearly demarked by extensive Ki67 expression (proliferative phenotype) and almost complete loss of CK19 immunoreactivity (lack of progenitor/biliary component), see Fig. 2G–I. Parenchymal (hepatocytes and inflammatory cells) expression of Ki67 and CK19 increased progressively with most marked increments observed after 60 weeks of GAN diet feeding (Fig. 2J–N). A subset of tumors were selected for further detailed microscopical evaluation. Five out of seven tumors were confirmed HCC and subsequently classified using the WHO three-tiered grading system based on a combination of cytological features and differentiation (G1–G3)^30^. Four out of five HCCs were moderately differentiated (G1 low grade, n = 1; G2 intermediate-grade, n = 4). Tumors not meeting criteria for HCC (2 out of 7 tumors) appeared micronodular with relatively well-preserved reticulin trabecular framework, suggestive of focal nodular hyperplasia (FNH). Tumors did not demonstrate morphological or histological features of adenomas or dysplastic nodules.

**Figure 3:**
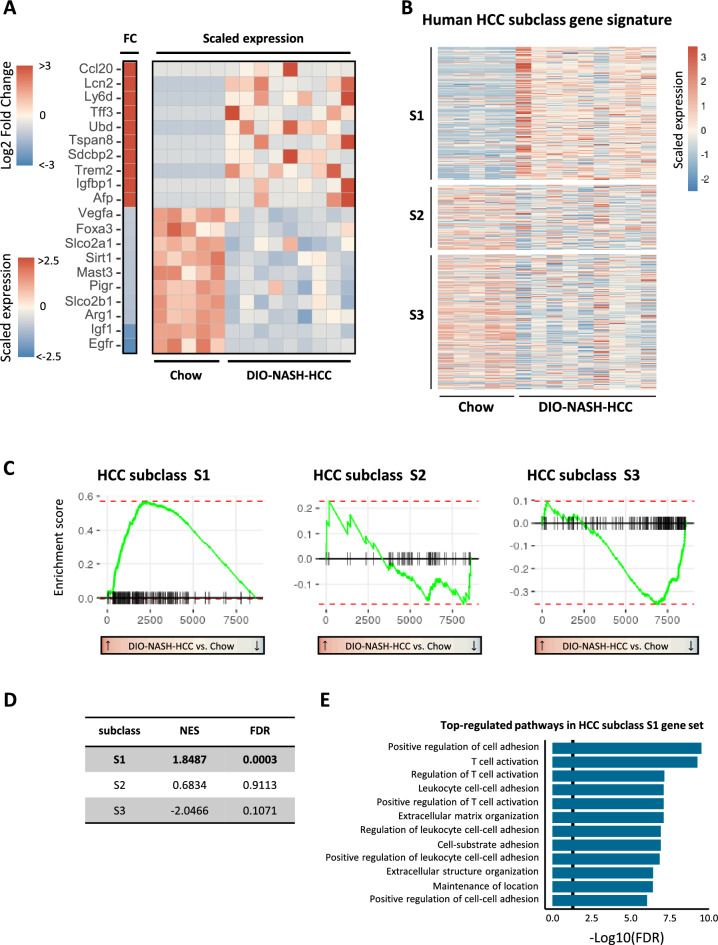
Molecular tumor signatures in GAN DIO-NASH-HCC mice. (**A**) Heatmap depicting top-10 upregulated and downregulated candidate oncogenes and tumor suppressor genes associated with human HCC in tumors from GAN DIO-NASH-HCC mice (n = 9) compared to healthy liver tissue samples from chow-fed controls (n = 5). Log2-fold change, false discovery rate < 0.05. See Fig. S4 for all 148 HCC-associated genes analyzed. Left heat map (vertical bar): Log2-fold change (FC) in gene expression in GAN DIO-NASH-HCC mice compared to chow controls (red color, significantly upregulated gene; blue color, significantly downregulated gene; false discovery rate *p* < 0.05). Right heatmap: Scaled gene expression for chow-fed mice and GAN DIO-NASH-HCC mice (red color, increased expression; blue color lower expression). (**B**) Human HCC subclass gene signature in tumors from GAN DIO-NASH-HCC mice (n = 9) compared to normal liver tissue samples from chow-fed mice (n = 5). Red color, increased expression; blue color, lower expression. Heatmap is depicting scaled expression of genes classifying human HCC molecular subclass S1 (210 genes), S2 (103 genes) and S3 (225 genes). (**C**) Comparative gene set enrichment analysis on isolated tumors for classification according to human HCC subclass S1–S3 molecular signatures. Left-ward curve shift (i.e. highest gene expression level in GAN DIO-NASH-HCC mice compared to chow-fed controls) indicates good concordance between human HCC S1 molecular subclass and gene expressing signatures in liver tumors from GAN DIO-NASH-HCC mice. (**D**) Normalized enrichment score (NES) indicating significant enrichment of upregulated tumor genes in GAN DIO-NASH-HCC tumors associated with human HCC S1 molecular subclass. (**E**) Functional Gene Ontology enrichment analysis on signaling pathway perturbations (top-12 listed pathways according to statistical significance, *p* < 0.05 indicated as vertical line in plot) in GAN DIO-NASH-HCC mouse tumors compared to chow-fed controls (normal liver tissue).

Hepatic non-tumorous tissue transcriptome signatures in GAN DIO-NASH-HCC mice markedly differed from chow controls (Fig. S3, panels A-B). In support of histological data, GAN DIO-NASH-HCC mice demonstrated widespread regulations in candidate gene sets linked to NASH and fibrosis, indicating defective handling of lipids, carbohydrates and bile acids in addition to stimulated immune activity and fibrogenesis (Fig. S3, panel C). Isolated tumors from GAN DIO-NASH-HCC mice demonstrated an extensive number of differentially expressed genes (DEGs) compared to corresponding adjacent non-tumorous (ANT) liver tissue samples (1249 DEGs) as well as compared to healthy liver samples from chow-fed controls (8637 DEGs). Next, we investigated human HCC-related oncogene and tumor suppressor gene markers in tumor samples from GAN DIO-NASH-HCC mice. A curated list of 148 mouse orthologs ofhuman HCC-related oncogenes were probed in tumors from GAN DIO-NASH-HCC mice. The major proportion of these genes were upregulated in tumors as compared to healthy livers samples from chow-fed controls (Figs. 3A, S4). The oncogene expression signature in tumors was distinct from ANT samples (Fig. S5). Molecular HCC subclassification was subsequently assessed according to subclass S1-S3 correlating with histological and clinical features of HCC^34^. Compared to chow-fed controls, liver tumors in GAN DIO-NASH-HCC mice demonstrated distinct enrichment of activated genes associated with human HCC subclass S1 (Wnt/TGFβ-proliferation, Fig. 3B), which has been associated with poor prognosis in HCC patients^34^. Tumors showed considerably fewer upregulated genes within subclass S2 (progenitor cell proliferation) and subclass S3 (non-proliferation). A gene set enrichment analysis (GSEA), based on preranked tumor-regulated genes (according to log2-fold change compared to chow-fed controls), supported activated tumor genes being overrepresented in the HCC S1 subclass (Fig. 3C). A normalized enrichment score (NES) was calculated based on S1-S3 molecular subclass genes significantly regulated in GAN DIO-NASH-HCC mouse tumors. Compared to chow-fed controls, significant enrichment of activated genes (+ 1.84, *p* = 0.0003) was only observed for S1 subclass genes (Fig. 3D). A signaling pathway analysis on activated HCC subclass S1 genes suggested perturbation of several signaling pathways involved in the regulation of immune system, with notable changes in T-cell signaling (Fig. 3E). As S1 subclass HCC is associated with the activation of Wnt-β-catenin pathway^34^, we performed single sample gene set enrichment analysis (ssGSEA)^35^ in tumors and corresponding ANT. Indeed, we observed significant upregulation of pathways associated with β-catenin dynamics (Fig. S6, panels A-E), as well as increased expression of several β-catenin target genes^36^ (Fig. S6, panel F). Similar to GAN DIO-NASH-HCC mouse tumors β-catenin-activated human HCCs do often not display steatosis, perhaps due to fatty acid β-oxidative reprogramming to support tumor growth^37^.

As we subsequently profiled semaglutide and lanifibranor treatment effects in GAN DIO-NASH-HCC mice (see below), hepatic gene expression levels of their cognate molecular targets were assessed in the model. *Glp1r* expression was not detected in tumor and ANT samples of GAN DIO-NASH-HCC mice. Both tissue types expressed *Ppara*, *Ppard* and *Pparg* (Fig. S7, panel A). Compared to chow controls, *Ppara* expression was significantly down-regulated in ANT, *Ppard* not regulated, and *Pparg* upregulated in ANT (Fig. S3, S7 panel A). No change was observed in tumor vs. ANT expression of the obligatory PPAR partners, retinoid X receptor subtypes *Rxra* and *Rxrb.* However, *Rxra* was downregulated in tumors compared to chow controls and *Rxrg* was upregulated in ANT compared to chow controls. Overall, we did not observe difference in the enrichment of PPAR signaling markers (Fig. S7, panel B). Only few PPAR-RXR targets showed significant difference in their expression between tumor and ANT, possibly pointing towards altered lipid metabolism and gluconeogenesis in HCC tumors (Fig. S7, panel C).

### Differential therapeutic profile of semaglutide and lanifibranor monotherapy in GAN DIO-NASH-HCC mice

**Figure 4:**
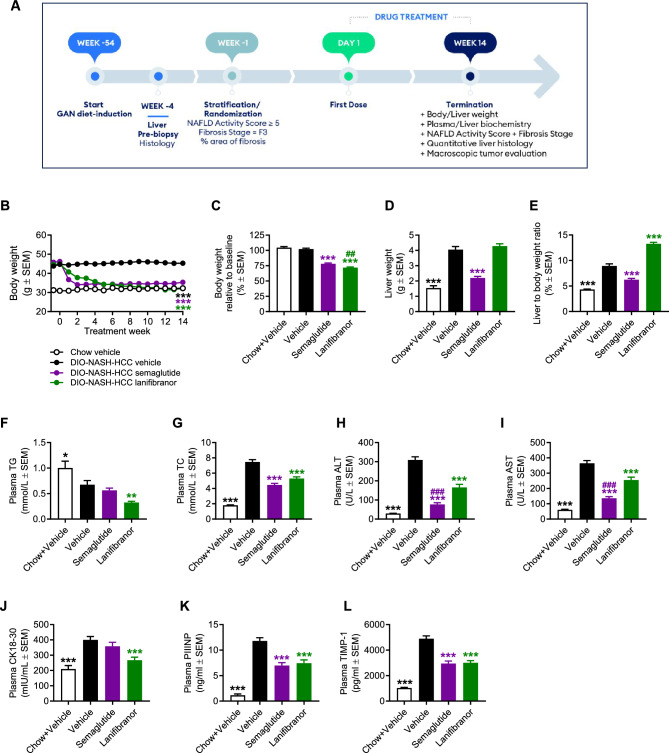
Semaglutide and lanifibranor reduce body weight and improve plasma transaminases in GAN DIO-NASH-HCC mice. GAN DIO-NASH-HCC mice with biopsy-confirmed NASH and fibrosis were administered (QD) vehicle (SC), semaglutide (30 nmol/kg, SC) or lanifibranor (mg/kg, PO) for 14 weeks (n = 15–16 per group). Treatment was initiated after 54 weeks of GAN diet feeding. Mice were stratified/randomized to treatment according to severity of NASH (NAS ≥ 5) and fibrosis (fibrosis stage F3) assessed 4 weeks before treatment start. Chow-fed mice receiving (QD) saline vehicle for 14 weeks (Chow + Vehicle) served as normal controls (n = 10). (**A**) Study outline. (**B**) Body weight curves over the entire treatment period. (**C**) Terminal body weight. (**D**) Liver weight. (**E**) Liver to body weight ratio (% of body weight). (**F**) Plasma triglycerides (TG). (**G**) Plasma total cholesterol (TC). (**H**) Plasma alanine transaminase (ALT). (**I**) Plasma aspartate transaminase (AST). (**J**) Plasma cytokeratin CK18-M30. (**K**) Plasma amino-terminal propeptide of type III procollagen (PIIINP). (**L**) Plasma tissue inhibitor of metalloproteinase-1 (TIMP-1). **p < 0.01, ***p < 0.001 versus vehicle-dosed GAN DIO-NASH mice, ^##^*p* < 0.01, ^###^*p* < 0.001 versus semaglutide/lanifibranor (Dunnett’s test one-factor linear model).

**Figure 5:**
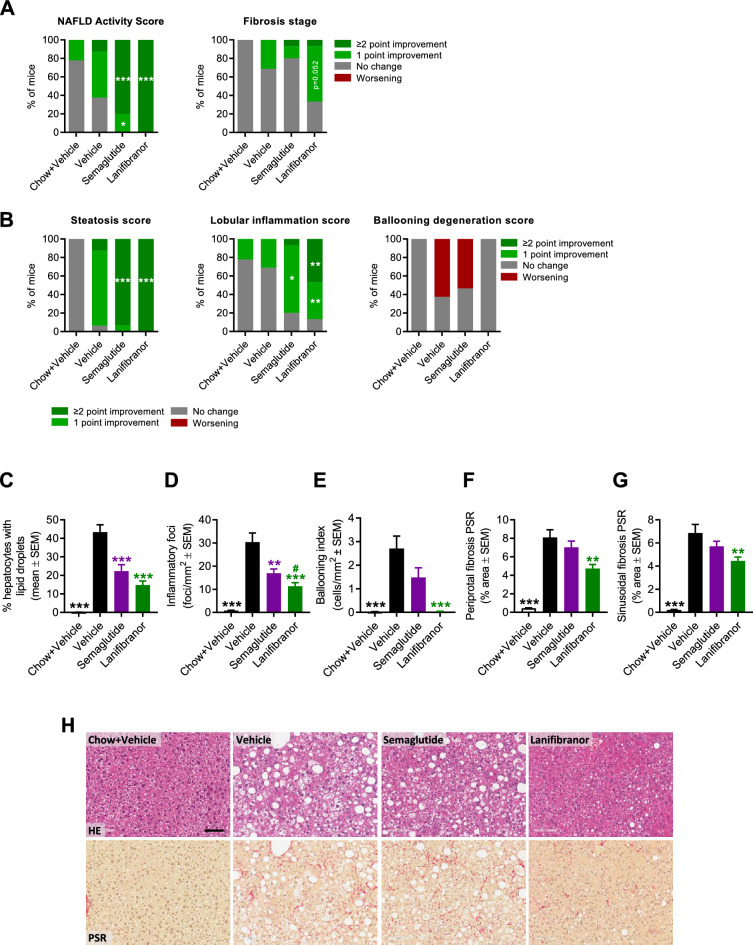
Semaglutide and lanifibranor differentially improves NASH histological hallmarks in GAN DIO-NASH-HCC mice. GAN DIO-NASH-HCC mice with biopsy-confirmed NASH and fibrosis were administered (QD) vehicle (SC), semaglutide (30 nmol/kg, SC) or lanifibranor (mg/kg, PO) for 14 weeks (n = 15–16 per group). Treatment was initiated after 54 weeks of GAN diet feeding. Mice were stratified/randomized to treatment according to severity of NASH (NAS ≥ 5) and fibrosis (fibrosis stage F3) assessed 4 weeks before treatment start. Chow-fed mice receiving (QD) saline vehicle for 14 weeks (Chow + Vehicle) served as normal controls (n = 10). (**A**) NAFLD Activity Score (NAS) and fibrosis stage. (**B**) Steatosis score, lobular inflammation score and ballooning degeneration score. *p < 0.05, **p < 0.01, ***p < 0.001 (one-sided Fisher’s exact test with Bonferroni correction). See Fig. S8 for changes in histopathological scores in individual mice. (**C-G**) Histomorphometric assessment of histopathological scoring variables as determined by the AI-based GHOST application. (**C**) Proportionate area (%) of hepatocytes with lipid droplets. (**D**) Number of inflammatory foci per mm^2^. (**E**) Hepatocyte ballooning (cells/mm^2^). (**F**, **G**) % area of sinusoidal fibrosis and periportal fibrosis. **p < 0.01, ***p < 0.001 vehicle-dosed GAN DIO-NASH mice; ^#^*p* < 0.05 versus semaglutide (Dunnett’s test one-factor linear model). (**H**) Representative photomicrographs illustrating reduced steatosis (HE staining) after semaglutide and lanifibranor treatment. Scale bar, 100 µm.

**Figure 6:**
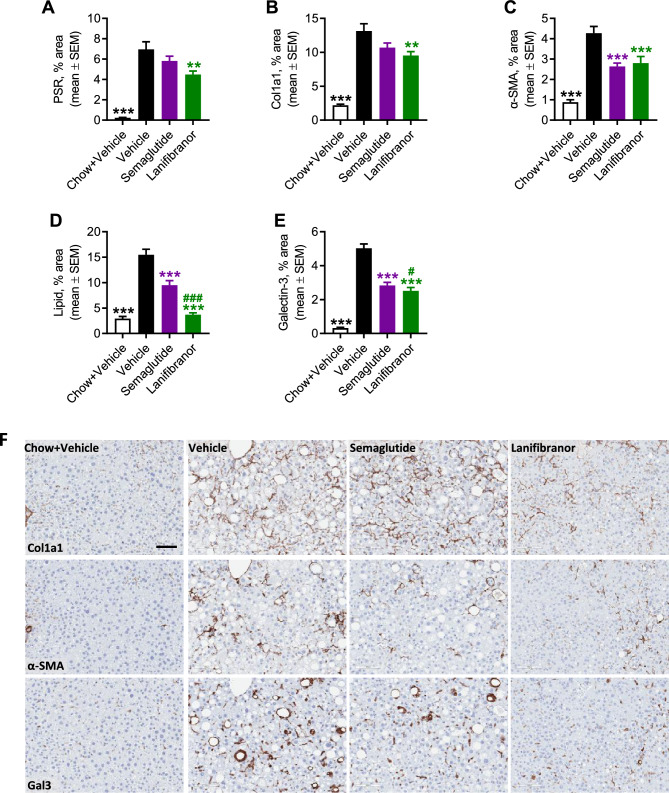
Semaglutide and lanifibranor differentially improves quantitative histological markers of fibrosis, steatosis and inflammation in GAN DIO-NASH-HCC mice. GAN DIO-NASH-HCC mice with biopsy-confirmed NASH and fibrosis were administered (QD) vehicle (SC), semaglutide (30 nmol/kg, SC) or lanifibranor (mg/kg, PO) for 14 weeks (n = 15–16 per group). Treatment was initiated after 54 weeks of GAN diet feeding. Mice were stratified/randomized to treatment according to severity of NASH (NAS ≥ 5) and fibrosis (fibrosis stage F3) assessed 4 weeks before treatment start. Chow-fed mice receiving (QD) saline vehicle for 14 weeks (Chow + Vehicle) served as normal controls (n = 10). (**A**–**E**) Proportionate (%) area of (**A**, **B**) Fibrosis (PSR, Col1a1), (**C**) α-SMA (fibrogenesis marker), (**D**) lipids, and (**E**) inflammation (galectin-3). (**F**) Representative photomicrographs illustrating reduced fibrosis (Col1a1), fibrogenesis (α-SMA) and inflammation (galectin-3) after semaglutide and lanifibranor treatment. Only lanifibranor reduced quantitative levels of fibrosis (PSR, Col1a1). Scale bar, 100 µm. **p < 0.01, ***p < 0.001 versus vehicle-dosed GAN DIO-NASH mice; ^#^*p* < 0.05, ^###^*p* < 0.001 versus semaglutide (Dunnett’s test one-factor linear model).

Pharmacological treatment was initiated in GAN DIO-NASH-HCC mice with biopsy-confirmed severe NASH and advanced fibrosis (NAS ≥ 5; steatosis score 3, lobular inflammation score ≥ 2; fibrosis stage F3), see study outline in Fig. 4A. Compared to baseline, both compounds promoted a sustained and robust weight loss after 14 weeks of treatment, most pronounced for lanifibranor (semaglutide, 22.0 ± 1.4%, *p* < 0.001; lanifibranor, 28.9 ± 1.1%, Fig. 4B, C). A beneficial effect on hepatomegaly was only observed for semaglutide (Fig. 4D, E), whereas both compounds improved plasma markers of liver lipids, injury and fibrosis (Fig. 4F–L). Notably, semaglutide and lanifibranor markedly improved histological hallmarks of NASH, as indicated by a robust improvement of NAS (≥ 2-point, Figs. 5A, S8). A larger proportion of lanifibranor-treated GAN DIO-NASH-HCC mice exhibited even greater reduction in NAS (≥ 3-point improvement: lanifibranor, 14/15 mice; semaglutide 6/16 mice, Fig. S8). Benefits on NASH were largely driven by reductions in steatosis and lobular inflammation scores (Fig. 5B). Fibrosis scores were unaffected by semaglutide and lanifibranor treatment (Figs. 5A, S8), albeit lanifibranor demonstrated borderline statistical significance for improving fibrosis stage in GAN DIO-NASH-HCC mice (1 ≥ point improvement: vehicle 5/17 mice; semaglutide 3/15 mice [*p* = 1.000 vs. vehicle]; lanifibranor 10/15 mice [*p* = 0.052 vs. vehicle]). Both treatments significantly reduced number of lipid-laden hepatocytes and inflammatory foci (Fig. 5C, D), whereas only lanifibranor significantly reduced hepatocyte ballooning index (Fig. 5E), as well as proportionate area and total levels of sinusoidal and periportal fibrosis (Fig. 5F, G). The antifibrotic effect of lanifibranor was further supported by a significant reduction in whole-section PSR and Col1a1 staining (Figs. 6A, B, S9). Semaglutide and lanifibranor equally reduced %-area and total levels α-SMA, a marker of myofibroblast (hepatic stellate cell) activation (Figs. 6C, S9). Consistent with the marked benefits on steatosis and lobular inflammation scores, semaglutide and lanifibranor significantly reduced %-area and total levels of lipids and galectin-3 (Figs. 6D, E, S9).

### Semaglutide, but not lanifibranor, improves tumor burden in GAN DIO-NASH-HCC mice

**Figure 7:**
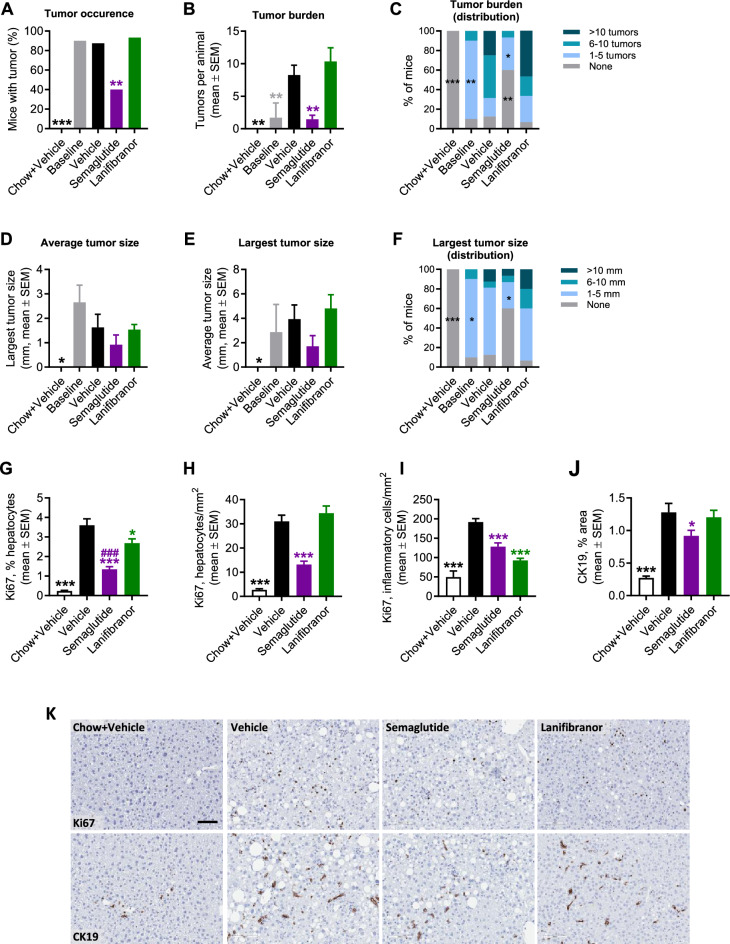
Semaglutide, but not lanifibranor, reduces tumor burden in GAN DIO-NASH-HCC mice. GAN DIO-NASH-HCC mice with biopsy-confirmed NASH were administered (QD) vehicle (SC), semaglutide (30 nmol/kg, SC) or lanifibranor (mg/kg, PO) for 14 weeks (n = 15–16 per group). Treatment was started after 54 weeks of GAN diet feeding. Chow-fed mice receiving (QD) saline vehicle for 14 weeks (Chow + Vehicle) served as normal controls (n = 10). Baseline HCC burden was assessed in a satellite group of GAN DIO-NASH-HCC mice (n = 10) after 54 weeks of GAN diet feeding. (**A**) Tumor occurence. (**B**) Tumor burden. (**C**) Distribution of tumor burden. (**D**) Average tumor size (mm). (**E**) Largest tumor size (diameter, mm). (**F**) Distribution of largest tumor size. *p < 0.05, **p < 0.01, ***p < 0.001 versus vehicle-dosed GAN DIO-NASH mice (Dunnett’s test one-factor linear model). (**G**–**I**) Ki67 staining of hepatocytes (relative number, %; area, mm^2^) and inflammatory cells (area, mm2). (**J**) Proportionate (%) area of CK19 staining. (**K**) Representative Ki67 and CK19 stainings illustrating treatment effects on Ki67 and CK19 staining. Scale bar, 100 µm. *p < 0.05, ***p < 0.001 versus vehicle-dosed GAN DIO-NASH mice; ^###^*p* < 0.001 versus lanifibranor (Dunnett’s test one-factor linear model).

A satellite group of GAN DIO-NASH mice was terminated to assess tumor incidence and burden at baseline. Nearly all GAN DIO-NASH mice (90%, 9 out of 10 mice) demonstrated macroscopically visible tumors (1.7 ± 0.8 tumors per mouse; average tumor size, 2.7 ± 0.7 mm; largest tumor size, 2.9 ± 0.7 mm, n = 10) after 54 weeks of GAN diet feeding (Fig. 7). Vehicle-dosed GAN DIO-NASH-HCC mice showed similar tumor incidence (88%, 14 out of 16 mice), however with a substantially greater tumor burden (8.3 ± 1.5 tumors per mouse), at termination after 72 weeks of GAN diet feeding (Fig. 7A–C). Compared to vehicle controls, semaglutide treatment for 14 weeks promoted a substantial reduction in HCC incidence (40%, 6 out of 15 mice, *p* < 0.01) and burden (1.5 ± 0.6 tumors per mouse, *p* < 0.01), see Fig. 7A–C. While semaglutide did not influence average tumor size (0.9 ± 0.4 mm, *p* = 0.430, Fig. 7D) and largest tumor size (1.7 ± 0.9 mm, *p* = 0.273, Fig. 7E), mice with tumors at study termination exhibited generally low tumor size after semaglutide treatment (Fig. 7F). In addition, semaglutide tended (*p* = 0.085) to reduce elevated plasma levels of alpha-fetoprotein (AFP), an oncofetal glycoprotein commonly used as circulating biomarker for HCC^38^, in GAN DIO-NASH-HCC mice (Fig. S10). In contrast, lanifibranor did not influence HCC incidence (93 ± 7%, 14 out of 15 mice, *p* = 0.583, Fig. 7A), burden (10.3 ± 2.1 tumors per mouse, *p* = 0.609, Fig. 7B, C), average tumor size (1.5 ± 0.2 mm, *p* = 0.997), largest tumor size (4.8 ± 1.1 mm, *p* = 0.871, Fig. 7E) or tumor size distribution (Fig. 7F) in GAN DIO-NASH-HCC mice. The anti-tumorigenic effect of semaglutide was accompanied by reduced density of Ki67-positive hepatocytes (Fig. 7G, H). Semaglutide also lowered the density of Ki67-positive inflammatory cells (Fig. 7I) and reduced %-area of CK19 staining (Figs. 7J, S9). A significant reduction in the relative number of Ki67-positive hepatocytes (Fig. 7G) and density of Ki67-positive inflammatory cells (Fig. 7I) was observed after lanifibranor treatment.

## Discussion

The present study establishes GAN DIO-NASH-HCC mice as a translational model of NASH-HCC with progressive tumor burden and molecular signatures of poor prognostic HCC. Therapeutic effects of long-term semaglutide and lanifibranor treatment on NASH and fibrosis histological endpoints in GAN DIO-NASH-HCC mice were overall comparable to primary outcomes reported in corresponding lage-stage clinical trials for NASH. It is noteworthy that semaglutide also improved HCC burden in GAN DIO-NASH-HCC mice, suggesting additional benefits of GLP1R agonists in the management of NASH complicated by HCC. Our study highlights the translatability and utility of GAN DIO-NASH-HCC mice for profiling novel drug therapies targeting fibrosing NASH and NASH-driven HCC.

Most animal models of NASH lack sufficient validation regarding disease progression. Here, we longitudinally profiled hepatic disease stages in GAN DIO-NASH mice, a translational model widely used in preclinical research and drug discovery for NASH^18–20^. The GAN diet promoted hepatic steatosis, inflammation and hepatocyte ballooning as assessed by automated deep learning-assisted image analysis (GHOST^20^) using clinical histopathological criteria outlined by Kleiner et al.^39^ Consistent with previous reports^18–20^, GAN DIO-NASH-HCC mice demonstrated histopathological hallmarks of NASH with a 100% incidence rate of steatosis, inflammation and fibrosis after approximately 6 months of GAN diet feeding. In contrast to increasing severity of steatosis and lobular inflammation, the hepatocyte ballooning component remained mild and was only detected in a subset of mice in the cohort, albeit becoming more prevalant with advancing disease. Accumulating evidence suggests that migration of immune cells into the liver plays a critical role in initiation and propagation of liver inflammation and fibrosis in NASH^40^. In particular, dynamic changes in infiltrating and resident hepatic macrophage populations is likely essential in NASH pathogenesis^41,42^. In concordance, changes in hepatic immune cell populations in GAN DIO-NASH-HCC mice were dominated by infiltrating inflammatory monocytes/macrophages, resident Kupffer-like macrophages and dendritic-like cells, indicating that macrophage-driven inflammation is a general characteristic of this model. Interestingly, activated CD8+ cytotoxic T cells were upregulated in GAN DIO-NASH-HCC mice, which has been linked to HCC and impaired responses to immune therapies in NASH-HCC^12^. While hepatocyte ballooning ultrastructure in current mouse models of NASH remains to be defined and compared to NASH patients, hepatocyte ballooning in rodents do generally not meet human criteria for prominent ballooning, which could suggest species differences in hepatocyte morphological reponses to NASH-inducing insults^43–46^. Fibrotic injury progressed with extended GAN diet feeding resulting in development of advanced fibrosis (stage F3) in all mice at ≥ 60 weeks. Progressive changes in NASH and fibrosis severity was supported by quantitative histology, including morphometrics on steatosis and lobular inflammation scoring variables. Cirrhosis is the dominant liver-related factor for mortality in NASH patients^47^. While fibrotic lesions in GAN DIO-NASH-HCC mice did not progress to manifest cirrhosis, a pre-cirrhortic stage was suggested by spontaneous regression of steatosis which became evident at 72 weeks. We have previously reported progressive depletion of liver lipid stores in GAN-DIO-NASH-HCC mice fed the GAN diet for up to 88 weeks^20^. As steatotic features are often not recognizable in the cirrhotic phase in NASH patients (‘burn out’ NASH)^28^, this could imply disease progression towards a pre-cirrhotic state in GAN DIO-NASH-HCC mice. The exact mechanisms underlying liver fat depletion in late-stage NASH are unclear but have been linked to vascular changes, mitochondrial dysfunction and onset of a catabolic state^48–51^. Resistance to cirrhotic lesions is a general characteristic of ‘Western diet’-based mouse models of NASH, possibly explained by mice not living long enough to develop manifest cirrhosis or, alternatively, have high capacity to handle dietary lipids and carbohydrates in the context of sustained nutrient overload that could otherwise exhaust liver regenerative responses and thereby perpetuate cirrhotic injury^52^.

The GAN DIO-NASH-HCC mouse consistently presents with clinical histological hallmarks of progressive NASH, fibrosis and HCC burden following extended GAN diet feeding. In further support of clinical translatability of the model, HCC develops in the context of natural disease progression and do not require induction by a chemical carcinogen^44^. The natural disease progression profile in GAN DIO-NASH-HCC mice supports the concept that multiple ‘hits’ initiated and sustained by excess energy intake from dietary fat and simple sugars in addition to cholesterol is key background for NASH and late-stage complications of the disease^53,54^. In particular, increased consumption of saturated fats and fructose has been strongly linked to intrahepatic lipid accumulation, lipogenesis, insulin resistance, hepatocyte oxidative stress and inflammation which are drivers of fibrogenesis^55,56^. As chronic inflammation and fibrosis advances in NASH, hepatocyte regenerative capacity becomes compromised resulting in progressive hepatocyte death, triggering compensatory proliferative responses that increase susceptibility to HCC development^57^. Also, it has been speculated that fructose and its metabolites could be important nutrient components contributing to tumor initiation and metastasis by enhancing metabolic stress^58^. It is therefore noteworthy that GAN DIO-NASH-HCC mice showed spontaneous and progressive hepatic tumor development with clear histological features of HCC, lending further support to clinical translatability of the model. Tumor incidence in GAN DIO-NASH-HCC mice correlated with fibrosis severity, with the majority of mice (≥ 70%) showing macroscopic neoplastic lesions after ~ 60 weeks of GAN diet feeding. In addition to well-demarcated tumor appearance, cytological atypia (mild nuclear pleomorphism, increased nuclear-to-cytoplasmic ratio) and extensive depletion of reticulin trabecular framework, also reported previously in GAN DIO-NASH-HCC mice^20^, tumors displayed abnormal glutamine synthetase staining. Loss of reticulin trabecular organization and glutamine synthetase-staining are characteristic features to distinguish well-differentiated HCCs from precursor (premalignant) lesions in humans^28,29,59^. HCCs were typically intermediate-differentiated (G2 grade) using the WHO grading system for human HCC classification^30^, and exhibited a gene activation signature closely resembling human HCC molecular subclass S1 (Wnt/TGFβ-proliferation). As for subclass S2 (progenitor cell proliferation), HCC subclass S1 carries a poor prognosis in the clinic and particularly associated with non-cirrhortic NASH compared to other aetiologies of HCC^34,60^. A proliferating profile of HCCs was further emphasized by extensive Ki67 labeling of tumor cells compared to lower levels in the surrounding tissue. Compared to chow controls, non-tumorous tissue in GAN DIO-NASH mice exhibited significantly higher Ki67 expression, suggesting a hepatocytic pro-tumorigenic environment. While it should be noted that CK19 has been proposed as a prognostic marker for progenitor cell proliferation in both HCC and cholangiocarcinoma^61^, absence of biliary epithelia in tumors in GAN DIO-NASH-HCC mice was suggested by loss of CK19-immunoreactivity. In addition to histological features of HCC, we observed a significant increase in plasma AFP levels in GAN DIO-NASH-HCC mice. AFP is highly expressed by hepatoblasts and circulating AFP is the most universally used biomarker for HCC in the clinic^38^. The high HCC incidence rate in GAN DIO-NASH-HCC mice is comparable to other ‘Western’ diet-based mouse models of NASH^46,62–64^, also demonstrating similar HCC molecular subclass-specificity^60,62,63^. While a subset of tumors in GAN DIO-NASH-HCC mice demonstrated morphological and histological features of FNH, the second most prevalent benign liver tumor in humans^65^, hepatocellular adenomas and dysplastic nodules were not identified. Overall, tumors did not demonstrate collagen deposition while consistently being highly α-SMA immunoreactive, potentially reflecting activation of cancer-associated fibroblasts (CAFs), a heterogenous group of activated fibroblasts and a major component of the tumor stroma. Peri-tumoral HSC signaling and ECM remodeling is considered important mechanisms contributing to HCC progression by forming a prometastatic microenvironment facilitating cancer cell adhesion, growth and migration^66^. Although we did not observe invasion of blood vessels, enhanced α-SMA expression could also imply ensuing vascularization as α-SMA is also an early marker of vascular smooth muscle cell maturation^67^. Upregulation of pro-angiogenetic factors has been reported in NASH patients and rodent models of NAFLD/NASH^68–70^. Pathological neovascularization, fuelled by chronic inflammation and fibrosis, can facilitate and aggrevate metastasis and HCC development^71,72^. Inflamed (‘hot’) and noninflamed (‘cold’) HCC tumors and their individual molecular signatures have been associated with differential response to immune-checkpoint inhibitors. Specifically, conversion of HCCs from a ‘cold’ into ‘hot’ immunogenic tumor microenvironment may be critical to increase sensitivity to immunotherapies in HCC^73^. NASH patients have been reported less responsive to HCC immunotherapy which has been linked to aberramt cytoxic CD8^+^ T-cell activation^12^. It is therefore noteworthy that tumor gene expression signatures in GAN DIO-NASH-HCC mice indicated substantial perturbations in signaling pathways regulating T-cell activity. Although only assessed in non-tumorous tissue, we also detected expansions in hepatic cytoxic CD8^+^ T-cell, macrophage and dendritic cell populations in GAN DIO-NASH-HCC mice. Collectively, this could point to highly immunogenic tumor profiles in GAN DIO-NASH-HCC mice which could potentially be indirectly or directly targeted by drugs in late-stage development for NASH.

14 weeks of semaglutide and lanifibranor monotherapy robustly improved histological hallmarks in GAN DIO-NASH-HCC mice. In contrast, only lanifibranor promoted fibrosis regression as indicated by quantitative histology. While only indirectly supporting attenuation of fibrogenic activity, both semaglutide and lanifibranor significantly lowered α-SMA levels, Similar distinct histological benefits of semaglutide and lanifibranor monotherapy have recently been reported in GAN DIO-NASH mice without HCC (treatment start after 34–38 weeks of GAN diet feeding, fibrosis stage F1-F3)^20^, which is in close agreement with primary endpoint outcomes in corresponding clinical trials in NASH patients with fibrosis (stage F1-F3)^25,26^. The lack of antifibrotic efficacy of semaglutide in GAN DIO-NASH-HCC mice with features of precirrhosis is also consistent with a recent clinical trial on NASH-related precirrhosis/cirrhosis^74^. In the current study, lanifibranor tended to meet the fibrosis endpoint applied in clinical trials (≥ 1 point improvement in fibrosis, *p* = 0.052). It should be noted that the relative difference in response rate between GAN DIO-NASH-HCC mice treated with lanifibranor or vehicle, respectively, for 14 weeks (10/15 vs. 5/17 mice; 67% vs. 29%; *p* = 0.106) was comparable to GAN DIO-NASH mice receiving a similar lanifibranor or vehicle dosing regimen for 12 weeks (7/14 vs. 2/16 mice; 50% vs. 13%, *p* = 0.032)^20^.

Liver-directed mechanisms have been implicated in the hepatoprotective effects of lanifibranor, involving modulatory effects on hepatocyte lipid handling, macrophage polarization and HSC activity^75,76^. In contrast, the principal mechanism underlying semaglutide’s hepatoprotective effects remains incompletely understood. Several studies have reported lack of hepatic and hepatocyte GLP1R mRNA and protein expression in the mouse^77,78^, rat^79^, non-human primate^80^ and human^80,81^. In agreement, *Glp1r* was detected neither in hepatic tumors nor ANT in GAN DIO-NASH-HCC mice. A similar finding has been reported in the GAN DIO-NASH mouse^20^, suggesting that semaglutide does not directly target the liver. It has previously been speculated that hepatic GLP1R signals could potentially derive from infiltrating immune cells serving as potential targets for GLP1R agonists to attenuate liver inflammation^77,82^. In line with this hypothesis, a preliminary study in NASH patients has reported GLP1R immunoreactivity in hepatic monocytes and basolateral hepatocytes lining areas with steatosis^83^. In contrast, we did not find any evidence of GLP1R mRNA expression in liver biopsies from a larger NASH patient cohort study^18,84^. While the discrepancy may be explained by use of different methods, any functional relevance of hepatic GLP1R expression in the context of NASH remains to be established. In sum, hepatoprotective effects of GLP1R agonist are most likely owing to stimulation of extrahepatic GLP1R function known to afford appetite suppression, reduce adiposity and improve peripheral insulin sensitivity, thereby leading to overall benefits on liver health secondary to improved features of the metabolic syndrome^85–87^.

This is the first preclinical study to demonstrate that semaglutide, a drug in late-stage clinical development for NASH, can reduce tumor burden in a translational mouse model of NASH-HCC. The tumor suppressive effect of semaglutide was reflected by greater inhibition of hepatocyte cell proliferation compared to lanifibranor. Although both semaglutide and lanifibranor significantly lowered α-SMA levels, semaglutide had no effect on fibrosis histology in GAN DIO-NASH-HCC mice. As *Glp1r* expression was undetectable in tumors and surrounding non-tumorous tissue in GAN DIO-NASH-HCC mice, this rules out any potential intratumor GLP1R-associated effects of semaglutide, rendering it most likely that semaglutide lowered tumor burden by improving whole-body metabolism. While it is well-established that weight loss following intensive dietary intervention leads to improvements in liver histology in NASH patients^88^, the impact of dietary intervention and weight loss on NASH-associated HCC outcomes remains preliminary^89^. Considering the robust weight loss efficacy (≥ 20%) achieved with semaglutide and lanifibranor monotherapy in GAN DIO-NASH-HCC mice, this argues for body weight-independent tumor suppressive effects of semaglutide. In agreement, GLP1R agonists have been reported to reduce hepatocarcinogenesis in non-obese chemical carcinogen-induced mouse models of HCC, perhaps by limiting liver pro-tumorigenic metabolic factors such as hepatic insulin resistance, steatosis and inflammation^90,91^. In addition, semaglutide-induced restoration of circulating NK cell cytokine production has recently been proposed as a GLP1R-dependent immune regulatory mechanism improving metabolic outcomes in obesity and reduce cancer risk^92^.

Whereas semaglutide has been reported to promote weight loss in NASH patients^25,74^, lanifibranor slightly increases body weight in NASH patients compared to placebo^26^. The differential body weight regulatory effects of lanifibranor in NASH patients and GAN DIO-NASH-HCC mice is likely explained by species differences in the expression, distribution and function of human and mouse PPARs^93^. It is also noteworthy that lanifibranor did not improve HCC burden in GAN DIO-NASH-HCC mice while robustly improving histological hallmarks of NASH and fibrosis in the model. In contrast to semaglutide, the therapeutic effects of lanifibranor were not accompanied by improved hepatomegaly. It is well-estabished that PPAR-α agonists promote peroxisomal proliferation which can cause liver hypertrophy in rodents, but not humans^94^. Accordingly, stimulated PPAR-α function has been implicated in rodent-specific tumorigenicity of PPAR agonists^95^. In contrast, PPAR-ϒ agonists promote tumor growth arrest and decrease tumor burden in rodent models of HCC^96^. It may therefore be speculated that the PPAR-α stimulatory component of lanifibranor, a balanced PPAR-α/δ/ϒ agonist^75^, could preclude anti-neoplastic in GAN DIO-NASH-HCC mice. Future studies must aim to further define the molecular mechanisms underlying the differential impact of semaglutide and lanifibranor on HCC burden in GAN DIO-NASH-HCC mice.

## Conclusion

GAN-DIO-NASH-HCC mice spontaneously develop HCC on the background of progressive, severe liver fibrosis. This is the first preclinical study to demonstrate that semaglutide, a drug in late-stage clinical development for NASH, reduces hepatic tumor burden in a translational mouse model of NASH-HCC. The good clinical translatability highlights utility of the GAN DIO-NASH-HCC mouse profiling novel drug therapies targeting NASH-HCC.

## Methods

### Ethics

All experiments complied with the provisions of the Danish Animal Experiments Act and were approved by the Danish Animal Experiments Council (license #2013-15-2934-00784). All animal experiments conducted were approved by the internal Gubra Animal Welfare Body and were in full compliance with internationally accepted principles for the care and use of laboratory animals and conform to the Animal Research: Reporting of In Vivo Experiments (ARRIVE) guidelines.

### Animals

Male C57BL/6 J mice (5–6 weeks old) were from Janvier Labs (Le Genest Saint Isle, France) and housed in a controlled environment (12 h light/dark cycle, lights on at 3 AM, 21 ± 2 °C, humidity 50 ± 10%). Each animal was identified by an implantable subcutaneous microchip (PetID Microchip, E-vet, Haderslev, Denmark). Mice had ad libitum access to tap water and chow (3.22 kcal/g, Altromin 1324, Brogaarden, Hoersholm, Denmark) or Gubra Amylin NASH diet [GAN diet, 4.49 kcal/g, 40 kcal-% fat (of these 46% saturated fatty acids by weight), 22% fructose, 10% sucrose, 2% cholesterol; D09100310, Research Diets]. Mice were fed chow or GAN diet for up to 72 weeks. Animals were terminated by cardiac puncture under isoflurane anesthesia.

### Baseline liver biopsy

Animals underwent liver biopsy before treatment intervention, as described in detail previously^97^. Mice were anesthetized with isoflurane, a midline abdominal incision was made to expose the left lateral lobe, and a cone-shaped biopsy of ~ 50 mg liver tissue was collected. Cut surfaces were electrocoagulated using an electrosurgicial unit. Thereafter, the liver was returned to the abdominal cavity, the abdominal wall was sutured and the skin was stapled. Animals received 5 mg/kg carprofen prior to surgery and on post-operative day 1 and 2. Animals were single-housed after the procedure and allowed to recover for 4 weeks prior to treatment start.

### Treatment intervention

Animals were fed the GAN diet for 54 weeks before treatment start (Fig. 4A). Only DIO-NASH mice with biopsy-confirmed severe steatosis (score 3), lobular inflammation (≥ score 2) and advanced fibrosis (stage F3) were included, evaluated using standard clinical biopsy histopathological scoring criteria (see below). Mice were randomized and stratified to treatment based on baseline mean steatosis, lobular inflammation, fibrosis stage, and % area of fibrosis (PSR). GAN DIO-NASH-HCC mice received vehicle (SC, n = 16), semaglutide (30 nmol/kg, SC, n = 15) or lanifibranor (30 mg/kg, PO, n = 15) once daily for 14 weeks. The dose of semaglutide and lanifibranor, respectively, was selected based on previous reported studies in GAN DIO-NASH mice^20^ and related DIO-NASH mouse models^76,98^. A dose-escalation scheme was implemented to reduce expected initial effects of semaglutide treatment, as transient GLP1R-induced discomfort in rodents, including taste aversion and pica behavior, is typically observed within the first 2–3 days of treatment^99^. Therefore, the semaglutide dose was increased through daily increments (0.6–1.2–2.4–4.8–12–30 nmol/kg) for reaching the target dose on treatment day 6, thereafter being maintained for the remainder of the treatment period. Age-matched chow-fed mice receiving saline vehicle (n = 9, SC) served as normal controls. To assess tumor burden at baseline, a satellite group of GAN DIO-NASH-HCC mice (n = 10) were terminated after 54 weeks of GAN diet feeding. Animals were kept on the GAN diet throughout the drug treatment period. Vehicle and compounds were administered in a dosing volume of 5 ml/kg. Body weight was measured daily.

### Plasma biochemistry

Non-fasted terminal blood was sampled from the tail vein, kept on ice and centrifuged (5 min, 4 °C, 6000× *g*) to generate EDTA-stabilized plasma. Plasma lipids (triglycerides, total cholesterol) as well as markers of liver injury (alanine aminotransferase (ALT), aspartate aminotransferase (AST) and caspase-cleaved cytokeratin CK18 (CK18-M30) were determined as described previously^18,97^. Plasma markers of fibrosis included amino-terminal propeptide of type III procollagen (PIIINP, #CSB-E08095m, CusabBio, Houston, TX) and tissue inhibitor of metalloproteinase-1 (TIMP-1). PIIINP was determined according to the manufacturer’s instructions, TIMP-1 was assayed as described previously^18^. Alpha-fetoprotein was determined according to the manufacturer’s instructions (#MAFP00, R&D Systems, Minneapolis, MN).

### Liver histology

Baseline liver biopsy and terminal samples (both from the left lateral lobe) were fixed overnight in 4% paraformaldehyde. Liver tissue was paraffin-embedded and sectioned (3 µm thickness). Sections were stained with hematoxylin–eosin (HE), picro-sirius rRed (PSR, Sigma-Aldrich, Broendby, Denmark), anti-galectin-3 (cat. 125402, Biolegend, San Diego, CA), alpha-smooth muscle actin (α-SMA, cat. ab124964, Abcam, Cambridge, UK), anti-type I collagen (Col1a1, cat. 1310-01, Southern Biotech, Birmingham, AL), anti-Ki67 (Cat#14-5698-82, eBioscience, San Diego, CA) or anti cytokeratin-19 (CK19, cat#10712–1-AP, Proteintech, Rosemont, IL) using standard procedures^87,97^. An automated deep learning-based digital imaging analysis pipeline (Gubra Histopathological Objective Scoring Technology, GHOST) was applied to obtain more accurate and objective method for assessment of histopathological scores^20^, using the clinical NAFLD Activity Scoring (NAS) and fibrosis staging system according to NASH Clinical Research Network (CRN) scoring system as outlined by Kleiner et al*.*^39^ In addition, deep learning-based image analysis was applied to histopathological scoring variables for quantifying whole-section number of lipid-laden hepatocytes (% of hepatocytes with lipids droplets), number of inflammatory foci (foci per mm^2^), hepatocyte ballooning index (cells/mm^2^) as well as proportionate (%) area of perisinusoidal and periportal fibrosis, respectively. Additionally, quantitative histomorphometry was performed using a digital imaging software (Visiomorph®, Visiopharm, Hørsholm, Denmark) for the determination of whole-section liver fat (HE-staining), fibrosis (PSR, Col1a1), inflammation (galectin-3), hepatic stellate cell (HSC) activation (α-SMA), cell proliferation (Ki67) and progenitor cell/cholangiocyte activation (CK19), expressed relative (%) to total sectional area. The total number and individual size (mm^2^) of macroscopic surface tumors per animal was quantified. Histological classification of hepatic tumors was performed by an expert clinical histopathologist, using DIO-NASH-HCC mice fed the GAN for ≥ 68 weeks. For reticulin staining, slides were immersed in potassium permanganate solution, followed by sulfuric acid, oxalic acid, ferric ammonium sulfate solution, silver nitrate solution, formaldehyde solution, gold chloride solution, and sodium thiosulfate solution (#1.00251, Sigma-Aldrich, St. Louis, MO). Sections were rinsed with distilled water before immersion in each solution and dehydrated in graded ethanol and xylene before cover slipping. Glutamine synthetase staining was used to support differentation between normal liver architecture with pericentral staining and neoplastic liver with diffuse or no staining. HCCs were subsequently evaluated according to the WHO three-tiered grading system (G1-G3) based on cytological features and differentiation^30^.

### Flow cytometry

Pieces of approximately 150 mg were cut from randomly selected liver samples (medial lobe) from GAN DIO-NASH-HCC mice (72 weeks on GAN diet) and chow-fed controls (n = 10 per group) and stored overnight in RPMI + 10% FCS. Tissue was enzymatically digested with a Collagenase (1.5 mg/ml, Roche, Basel, Switzerland) and DNAse I (0.4 mg/ml; Roche, Basel, Switzerland) enzyme mix for 45 min. at 37 °C and sequentially passed through 100 µm and 60 µm filters to yield a single-cell suspension. Samples were blocked with anti-CD16/CD32 antibody TruStain fcX™ (Biolegend, San Diego, CA), incubated with the viability marker Zombie Aqua™ (BioLegend, San Diego, CA) and subsequently stained with one of two antibody panels to phenotype lymphoid cells [CD45 PE-Cy7 (clone I3/2.3), CD11b BV650 (clone M1/70), CD3 FITC (clone KT3.1.1), CD4 BV421 (clone GK1.5), CD8 APC (clone 53-6.7), NK1.1 PE (clone PK136), B220 BV605 (clone RA3-6B2), CD19 APC Fire 750 (clone 6D5) and CD25 PE-Dazzle 594 (clone PC61)] and myeloid cells [CD45 PE-Cy7 (clone I3/2.3), CD11b BV650 (clone M1/70), Ly6G BV605 (clone 1A8), Ly6C APC (clone HK1.4), F4/80 BV421 (clone T45-2342) and CD11c (clone N418)]. Prior the analysis cells were passed through a 40 µm filter, 50 µl of CountBright™ counting beads (Invitrogen, Carlsbad, CA) were added to each sample and flow cytometry was performed on a 4-laser CytoFlex S (Beckman Coulter, Indianapolis, IN). Data was analyzed using the CytExpert 2.2 software (Beckman Coulter, Indianapolis, IN).

### RNA sequencing and molecular classification of tumors

RNA sequencing was performed on terminal liver RNA extracts from GAN DIO-NASH-HCC mice (tumor samples, n = 9; ANT samples, n = 9) and chow-fed controls (healthy liver samples, n = 5) as described in detail elsewhere^18^. RNA sequence libraries were prepared using the NEBNext® Ultra™ II Directional RNA Library Prep Kit for Illumina (New England Biolabs, Ipswich, MA) and sequenced on NextSeq 500 (Illumina, San Diego, CA) with NextSeq 500/550 High Output Kit V2 (#75 CYS, Illumina). Reads were aligned to the GRCm38 v96 Ensembl Mus musculus genome using STAR v.2.7.0f.^100^ with default parameters. All RNA-seq data analyses were performed using the statistical software R^101^. Genes with at least 1 RPKM in a minimum of five samples (corresponding to the smallest group size), were kept for downstream analysis. The R-package DESeq2 v1.24.0^102^ was used for differential gene expression analysis, and p-values were corrected for multiple testing using the Benjamini–Hochberg method (5% False Discovery Rate, FDR < 0.05). Gene expression and regulation of a curated set of oncogenes and tumor suppressor genes (148 mouse homologues out of 158 human gene markers) previously reported associated with human HCC^31–33^ were investigated in isolated tumors and corresponding ANT tissue samples from GAN DIO-NASH-HCC mice. For classification of the tumor molecular signature, preranked gene set enrichment analysis (GSEA) was performed on all tumor-regulated genes against a reference gene set defining human HCC molecular subclasses^34^, i.e. S1 (208 mouse homologues out of 238 human gene markers), S2 (101 mouse homologues out of 113 human gene markers) and S3 (221 mouse homologues out of 262 human gene markers). GSEA analysis was performed using R-package fgsea v1.10.0^106^. Functional Gene Ontology enrichment analysis of upregulated subclass S1 genes was performed using the R-Package ClusterProfiler v3.12.0^107^. NASH and fibrosis-associated candidate genes^20^ were probed in non-tumorous tissue samples from GAN DIO-NASH-HCC mice compared to normal liver samples from chow-fed mice. Single sample gene set enrichment analysis (ssGSEA) using Reactome and WikiPathways datasets (v2023.2) was performed with GenePattern 2.0. Comparision between two groups for selected genes or pathways was performed with Mann–Whitney test and more than two groups was performed with Kruskall-Wallice non-parametric test.

### Statistics

Except from deep learning-based image analysis and RNA sequencing, data were analysed using GraphPad Prism v9.5.1 software (GraphPad, La Jolla, CA). All results are shown as mean ± standard error of mean (SEM). A one-sided Fisher's exact test with Bonferroni correction was used for within-subject comparison of histopathological scores before and after treatment intervention. A Dunnett’s test one- or two-factor linear model with interaction was used for all other statistical analyses. A *p*-value < 0.05 considered statistically significant.

### Supplementary Information

Supplementary Figures.

## Data Availability

The RNA sequencing datasets generated in the current study are available in the Gene Expression Omnibus (GEO) repository [https://www.ncbi.nlm.nih.gov/geo/; accession number GSE243976].
