# Supplementary material for: Semaglutide reduces tumor burden in the GAN diet-induced obese and biopsy-confirmed mouse model of NASH-HCC with advanced fibrosis
Source: Sci Rep. 2023 Dec 27;13:23056. doi: 10.1038/s41598-023-50328-5 (PMC10754821; doi:10.1038/s41598-023-50328-5)
Supplement: Supplementary file 1 — Supplementary Figures. [file 41598_2023_50328_MOESM1_ESM.pdf]

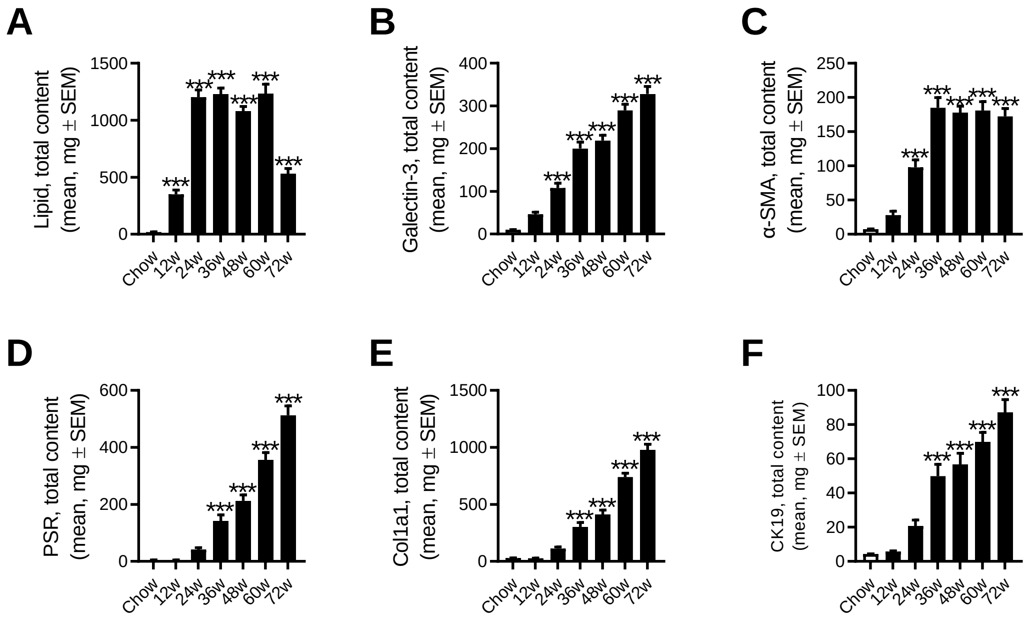

Figure S1. Progressive changes in liver histomorphometric markers in GAN DIO-NASH-HCC mice. Total liver histological marker levels of **(A)** lipids (HE staining), **(B)** inflammation (galectin-3), **(C)** hepatic stellate cell activation (α-SMA), **(D, E)** collagen (PSR, Col1a1), and **(F)** biliary/progenitor cell activation (CK19). To account for GAN diet-induced changes in liver mass, whole-liver marker content (mg per liver) was estimated by multiplying %-area of positive staining with corresponding total liver weight, being representative for whole-liver histological changes in DIO-NASH mice (Baandrup Kristiansen *et al.* BMC Gastroenterol 2019, 19, 228). GAN DIO-NASH-HCC mice were fed GAN diet for 12-72 weeks (n=15 per group), chow-fed mice (Chow) served as normal controls (n=10). ***p<0.001 vs. Chow (Dunnett's test one-factor linear model).

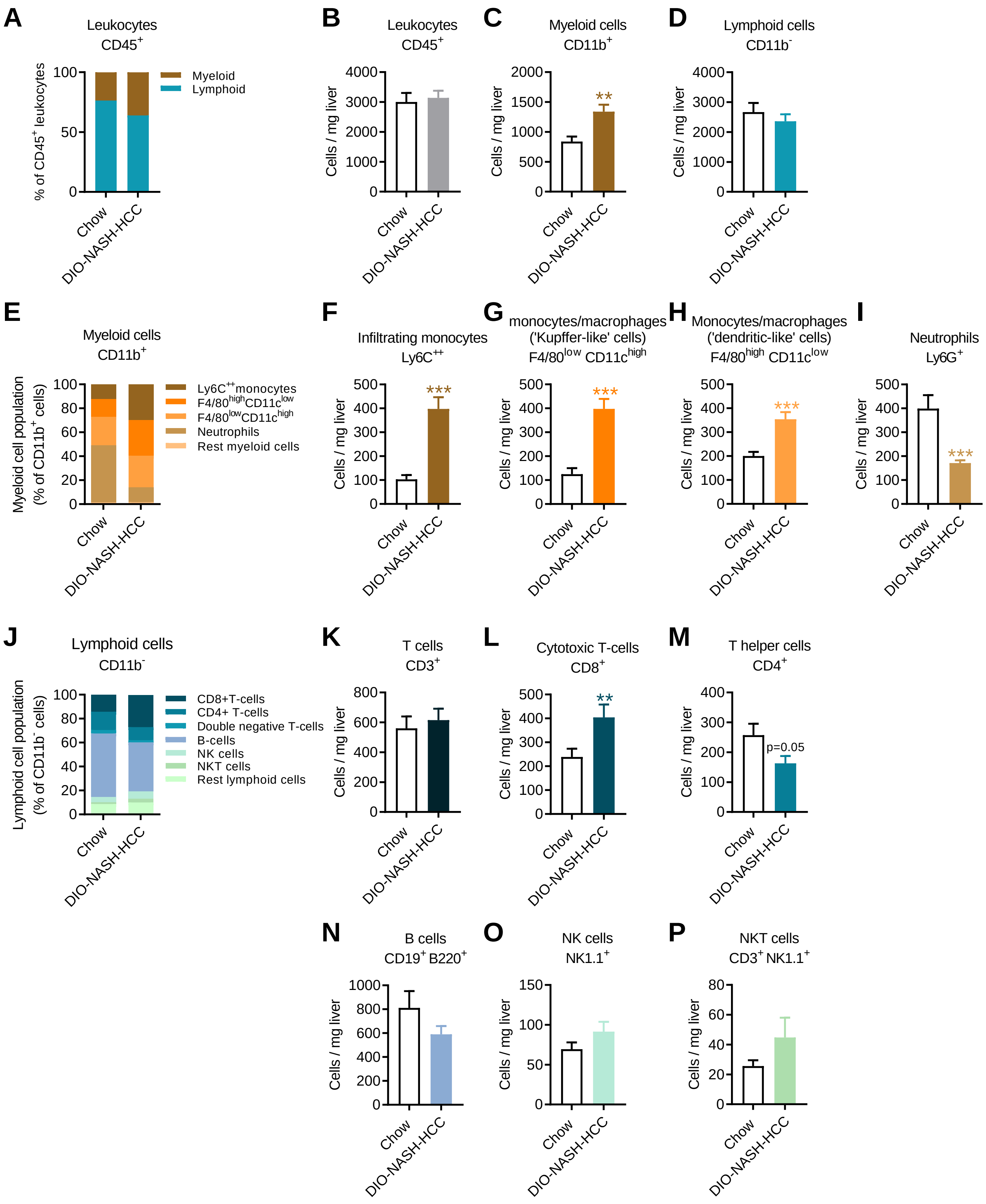

Figure S2. GAN DIO-NASH-HCC mice show marked expansions in hepatic myeloid immune cell subsets and cytotoxic T-cells. (A, B) Relative proportions (%, cells/mg) of leukocytes and lymphocytes. **(C-D)** Density (cells/mg) of myeloid and lymphoid cells. **(E)** Distribution of myeloid cell types. **(F-I)** Density (cells/mg) of infiltrating monocytes, Kupffer-like cells, dendritic-like cells and neutrophils. **(J)** Distribution of lymphoid cell types. **(K-P)** Density (cells/mg) of T-cells, cytotoxic T-cells, T-helper cells, B, cells, NK cells and NKT cells. GAN DIO-NASH mice were fed the GAN diet for 72 weeks (n=10). Chow-fed mice served as normal controls (n=10). **p<0.01, ***p<0.001 vs. Chow (Dunnett's test one-factor linear model).

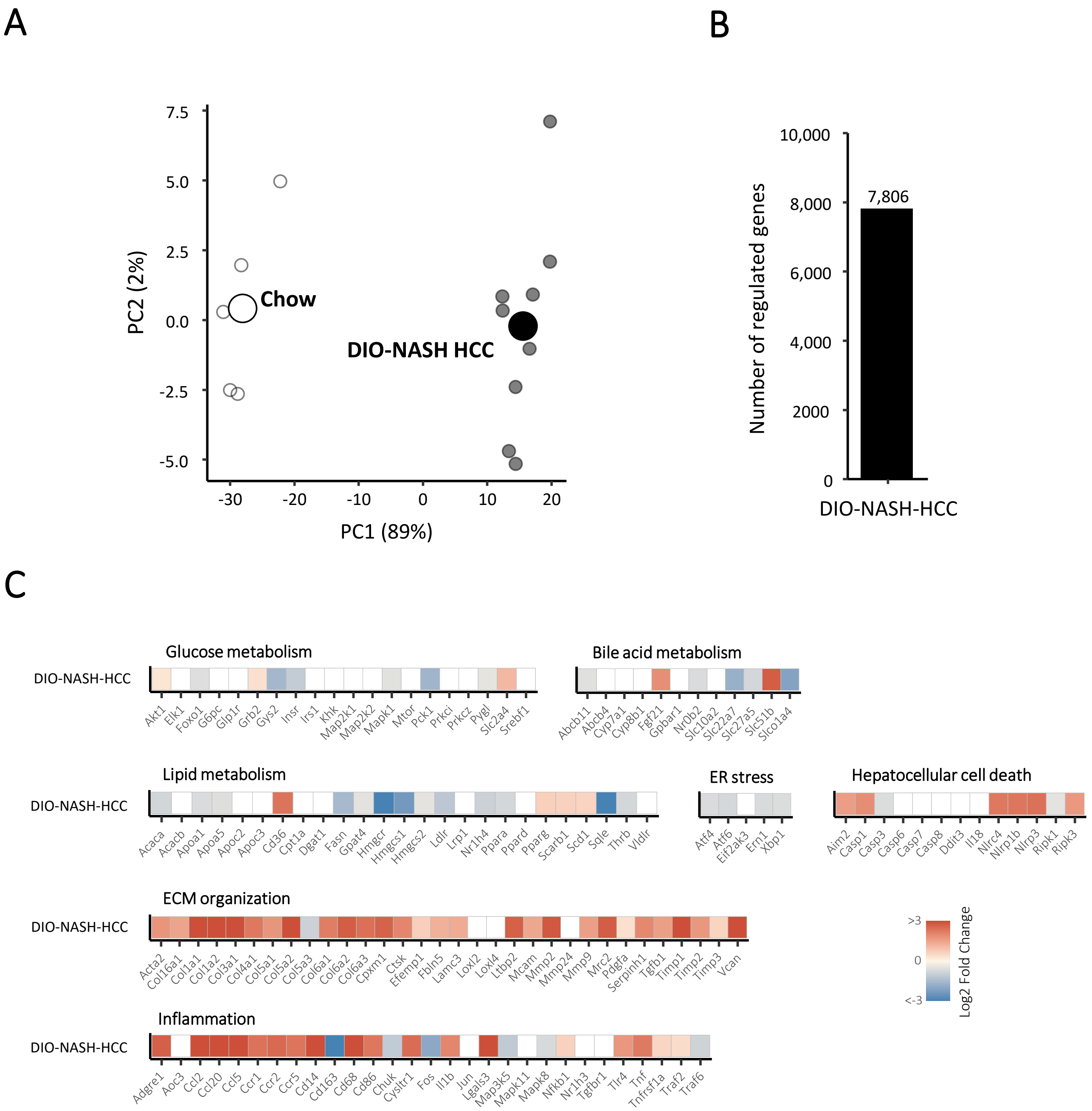

Figure S3. NASH-linked hepatic gene expression signatures in GAN DIO-NASH-HCC mice. Animals were fed GAN diet for 72 weeks. Age-matched chow-fed mice served as normal controls. All data are from RNA sequencing analysis on adjacent non-tumorous (ANT) tissue samples. **(A)** Principal component analysis (PCA) of samples based on top 500 most variable gene expression levels. **(B)** Total number of differentially expressed genes in GAN DIO-NASH-HCC mice compared to chow control. **(C)** Heatmaps illustrating changes in NASH and fibrosis-associated candidate gene expression in GAN DIO-NASH-HCC mice compared to chow-fed mice (log2-fold change). Color gradients in heatmaps indicate significant upregulation (red color), significant downregulation (blue color), or no significant change (white color) in gene expression (false discovery rate < 0.05).

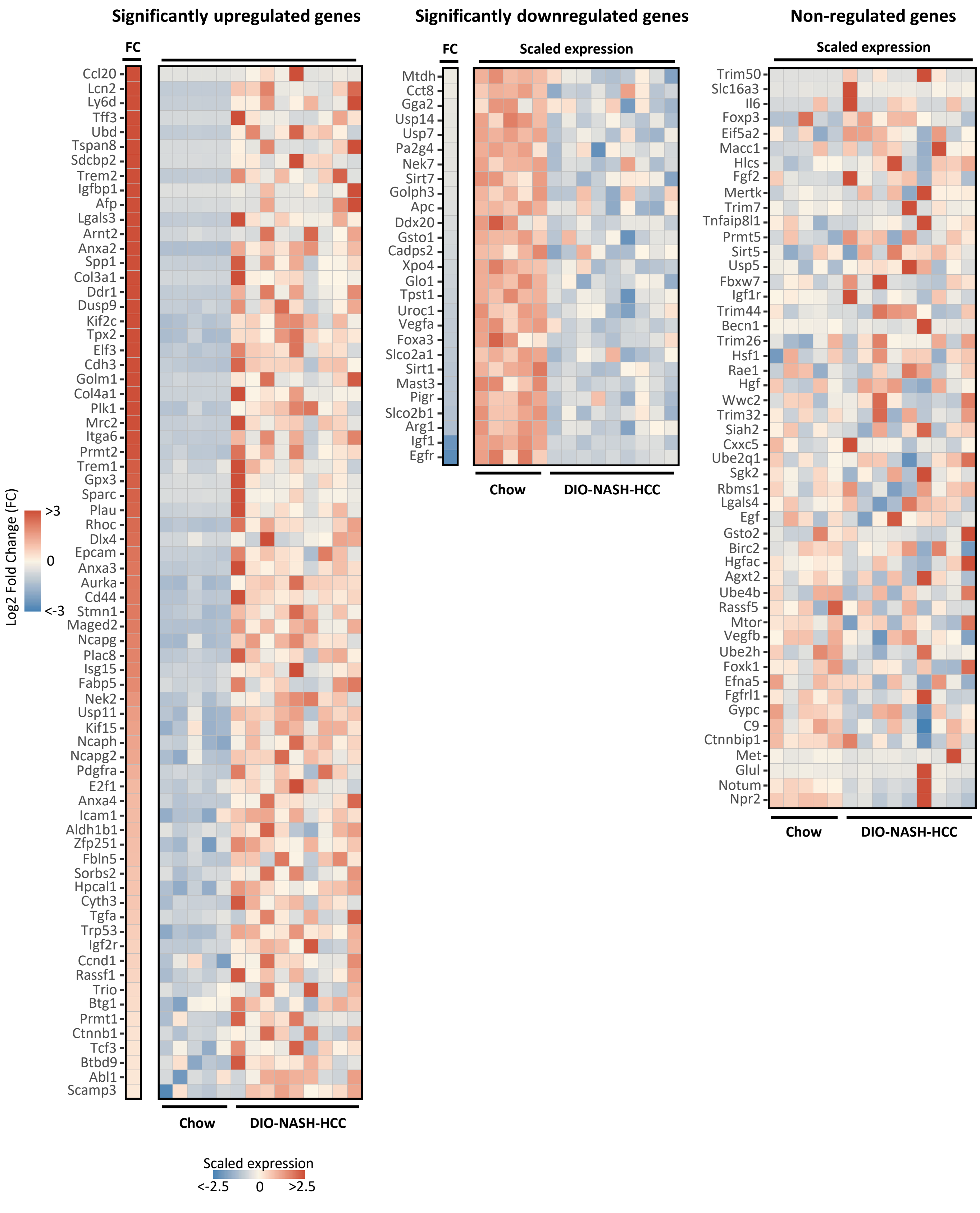

Figure S4. Heatmap on human HCC-associated oncogenes and tumor suppressor gene expression signatures in tumors from GAN DIO-NASH-HCC mice. Scaled gene expression in chow-fed mice (normal liver tissue, n=5) and GAN DIO-NASH-HCC mouse tumors (n=9). Red color, increased expression; blue color, lower expression. Left vertical bar indicates log2-fold change (FC) in gene expression in GAN DIO-NASH-HCC mice compared to chow controls (red bar, significant upregulation; blue bar, significantly downregulated; white bar, non-significant regulation). Significantly upregulated genes (n=71 genes), significantly downregulated genes (n=27 genes) and non-regulated genes (n=50 genes). False discovery rate < 0.05.

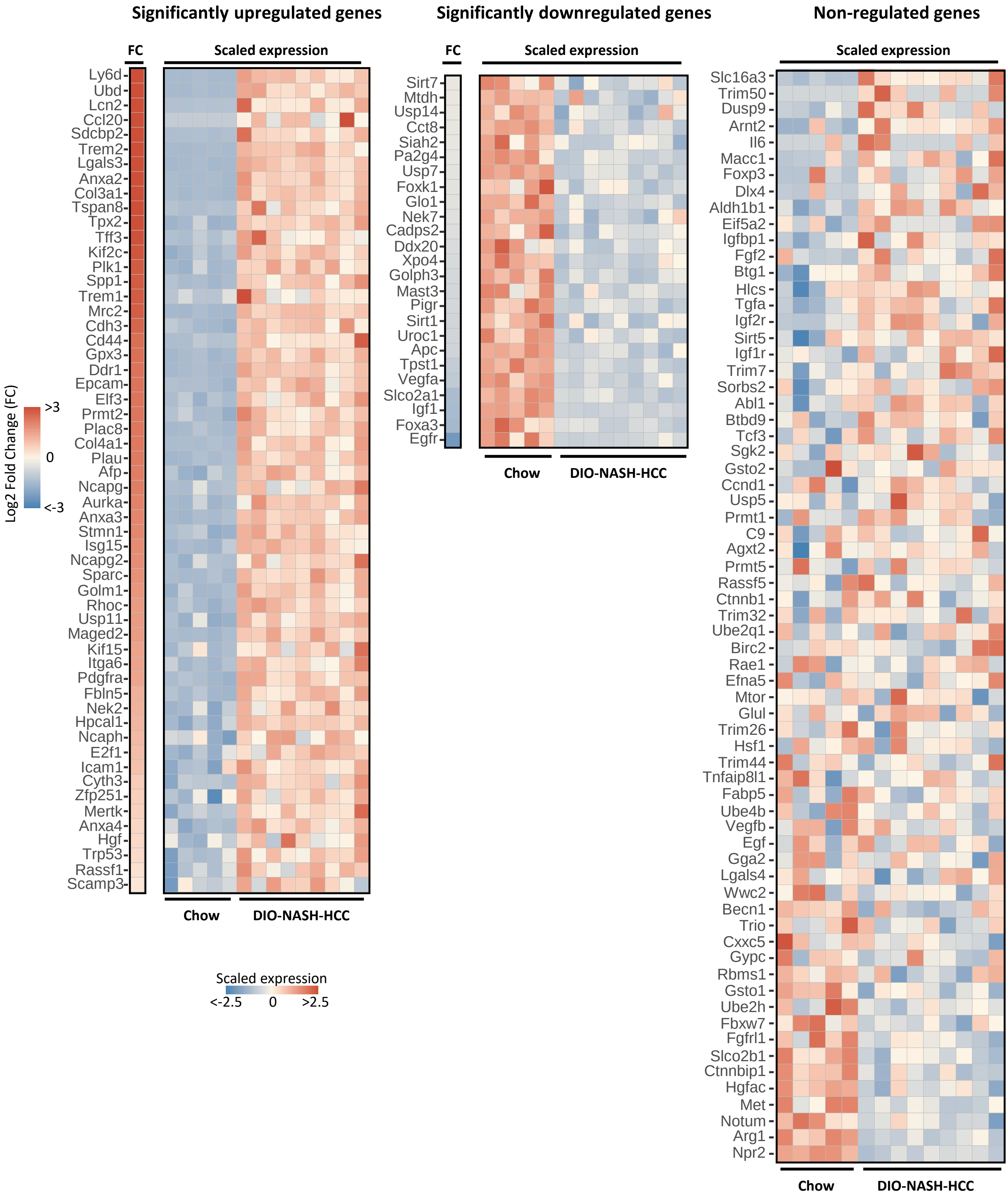

Figure S5. Heatmap on human HCC-associated oncogenes and tumor suppressor gene expression signatures in adjacent non-tumorous (ANT) liver tissue from GAN DIO-NASH-HCC mice. Scaled gene expression in chow-fed mice (normal liver tissue, n=5) and GAN DIO-NASH-HCC mouse adjacent non-tumorous liver tissue (n=9). Red color, increased expression; blue color, lower expression. Left vertical bar indicates log2-fold change (FC) in gene expression in GAN DIO-NASH-HCC mice compared to chow controls (red bar, significant upregulation; blue bar, significantly downregulated; white bar, non-significant regulation). Significantly upregulated genes (n=56 genes), significantly downregulated genes (n=25 genes) and non-regulated genes (n=67 genes). False discovery rate < 0.05.

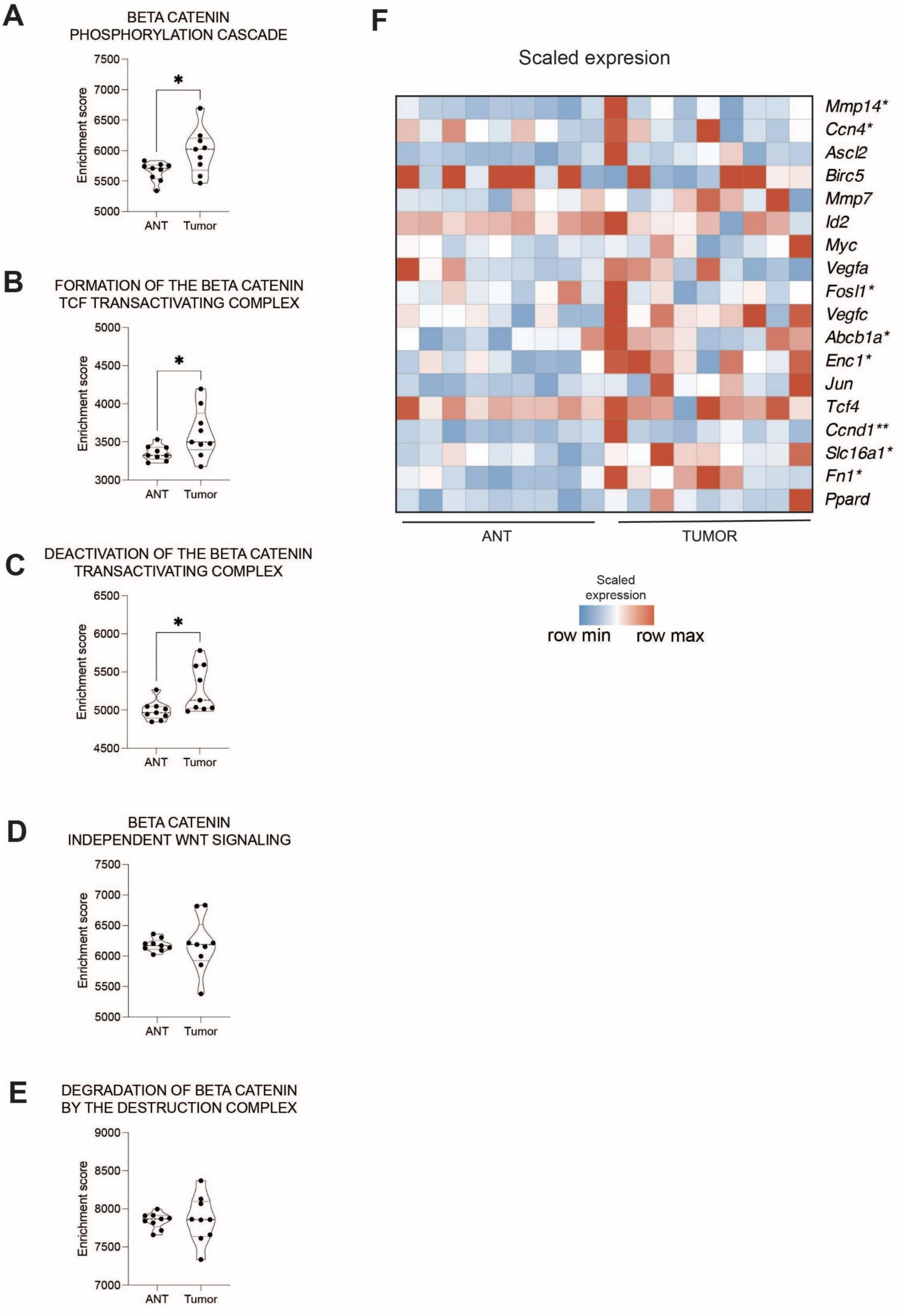

Figure S6. Beta-catenin signaling is activated in GAN-DIO-NASH-HCC tumors compared to adjacent non-tumorous (ANT) liver tissue. (A-E) ssGSEA enrichment of β-catenin associated pathways in GAN-DIO-NASH-HCC mice, *p<0.05. **(F)** Heatmap presenting z-scored expression of β-catenin target genes. Mann-Whitney U test, *p<0.05, **p<0.01.

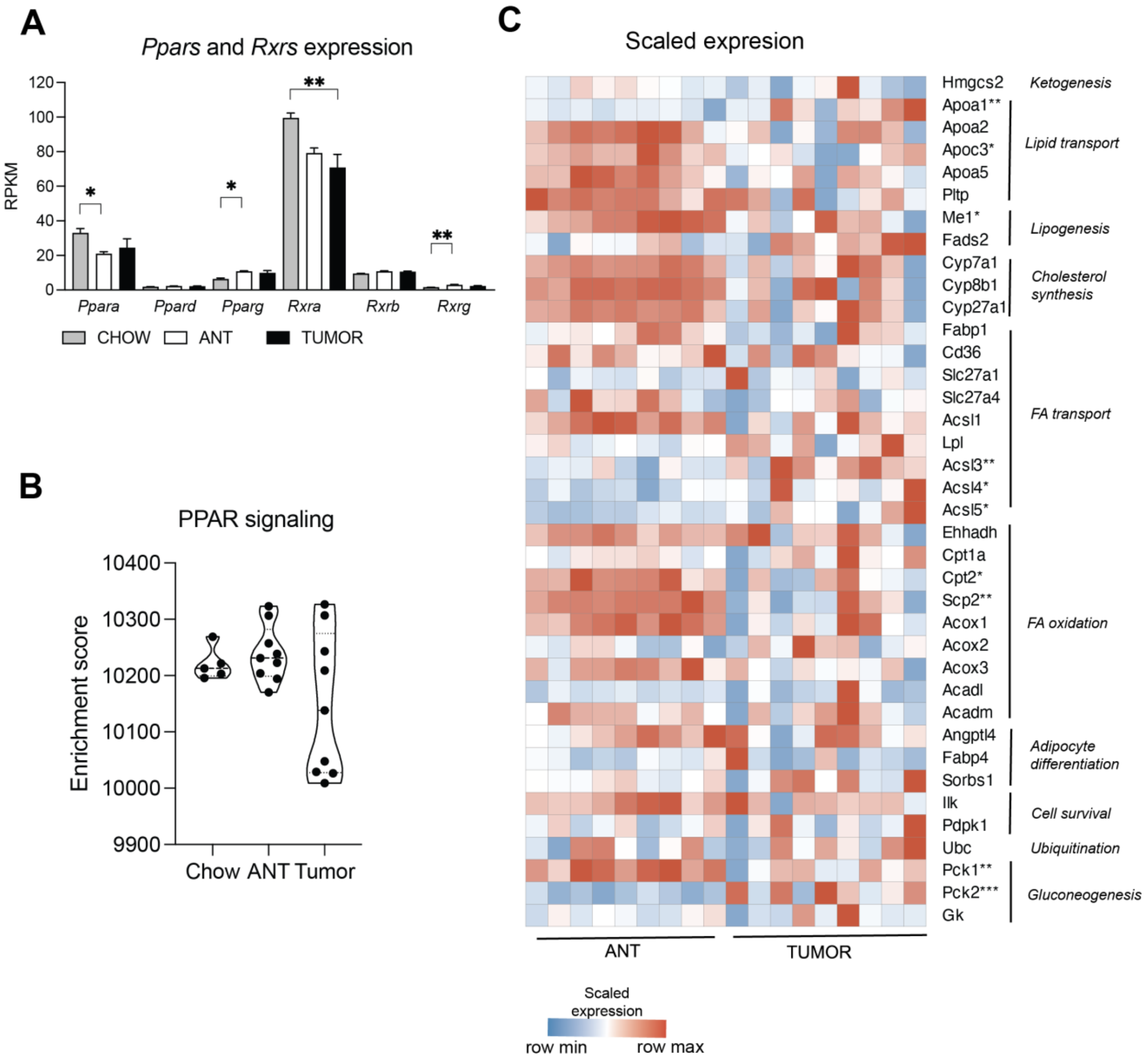

Figure S7. Alterations in PPAR signaling pathway in GAN-DIO-NASH-HCC tumors.
(A) Expression of *Ppar* and *Rxr* genes in tumor and adjacent non-tumorous tissue (ANT) in GAN-DIO-NASH-HCC mice (n=9) Kruskal-Wallice non-parametric test between 3 groups *p<0.05, **p<0.01. **(B)** The violin plot representing enrichment scores for single sample gene-set enrichment analysis for PPAR signaling pathway (Wiki Pathways), Kruskal-Wallice non-parametric test. **(C)** Heatmap representing PPAR transcription targets and associated signaling pathways. Mann-Whitney U test, *p<0.05, **p<0.01.

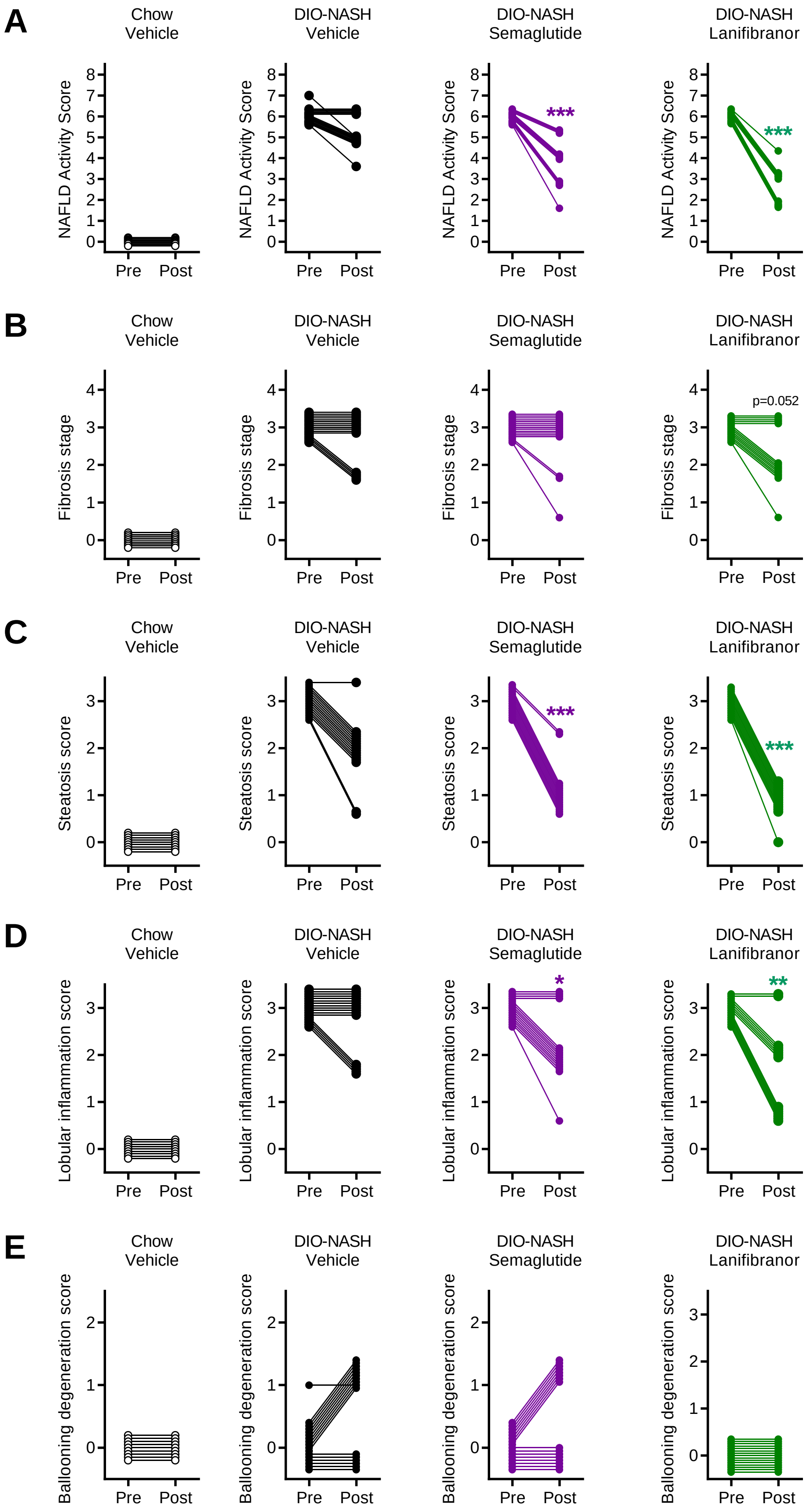

Figure S8. Effect of semaglutide or lanifibranor treatment on liver histopathological scores in GAN DIO-NASH-HCC mice. GAN DIO-NASH-HCC mice were administered (QD) vehicle (SC, n=16), semaglutide (30 nmol/kg, SC, n=15), or lanifibranor (30 mg/kg, PO, n=15) for 14 weeks. Vehicle-dosed (SC) chow-fed mice served as normal controls (n=9). Comparison of individual pre-post liver biopsy histopathological scores. **(A)** NAS (NAFLD Activity Score). **(B)** Fibrosis stage, **(C)** Steatosis score. **(D)** Lobular inflammation score. **(E)** Hepatocyte ballooning degeneration score **p<0.01, ***p<0.01, ***p<0.001 vs. DIO-NASH Vehicle control (one-sided Fisher's exact test with Bonferroni correction).

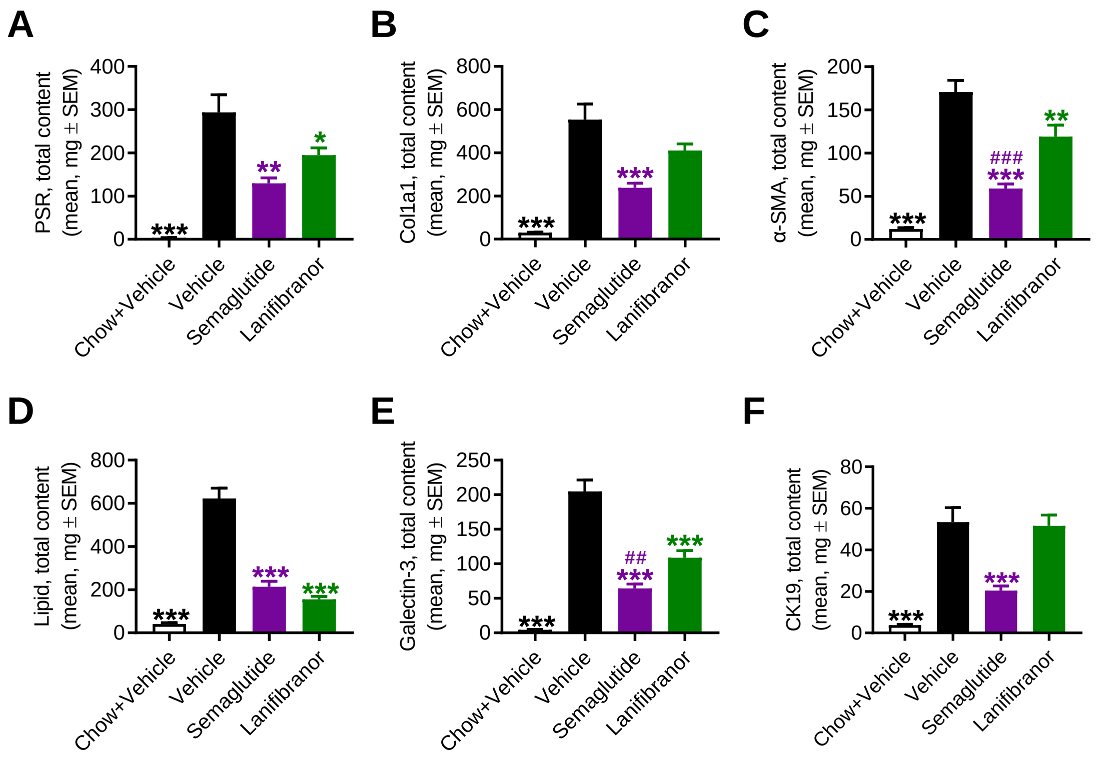

Figure S9. Semaglutide and lanifibranor differentially improves quantitative liver histological markers of NASH and fibrosis in GAN DIO-NASH-HCC mice. Total liver levels of **(A, B)** collagen (PSR, Col1a1), **(C)** active hepatic stellate cells (α-SMA), **(D)** lipids, **(E)** inflammation (galectin-3), and **(F)** active biliary/progenitor cells (CK19). To account for treatment-induced changes in liver mass, whole-liver marker content (mg per liver) was estimated by multiplying %-area of positive staining with corresponding total liver weight, being representative for whole-liver histological changes in DIO-NASH mice (Baandrup Kristiansen *et al.* BMC Gastroenterol 2019, 19, 228). GAN DIO-NASH-HCC mice were administered (QD) vehicle (SC, n=16), semaglutide (30 nmol/kg, SC, n=15), lanifibranor (30 mg/kg, PO, n=15) for 14 weeks. Chow-fed mice receiving (QD) saline vehicle (Chow + Vehicle) served as normal controls (n=9). *p<0.05, **p<0.01, ***p<0.001 vs. GAN DIO-NASH-HCC Vehicle controls, ##p<0.01, ###p<0.001 vs. lanifibranor (Dunnett's test one-factor linear model).

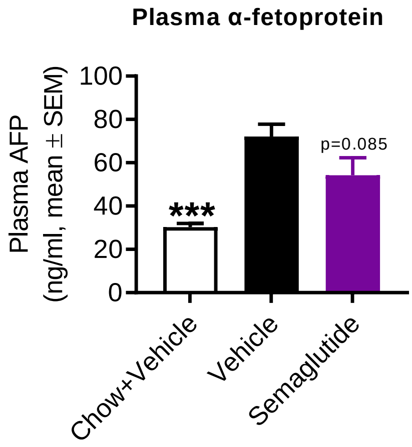

Figure S10. Semaglutide tends to reduce elevated plasma alpha-fetoprotein (AFP) levels in GAN DIO-NASH-HCC mice. Chow+Vehicle (n=9), GAN DIO-NASH-HCC Vehicle (n=15), GAN DIO-NASH-HCC semaglutide (n=14). ***p<0.001 vs. GAN DIO-NASH-HCC Vehicle group (Dunnett's test, one-factor linear model with interaction).
